# Embedding Pulmonary Rehabilitation for Chronic Obstructive Pulmonary Disease in the Home and Community Setting: A Rapid Review

**DOI:** 10.3389/fresc.2022.780736

**Published:** 2022-03-30

**Authors:** Túlio Medina Dutra de Oliveira, Adriano Luiz Pereira, Giovani Bernardo Costa, Liliane P. de Souza Mendes, Leonardo Barbosa de Almeida, Marcelo Velloso, Carla Malaguti

**Affiliations:** ^1^Department of Cardiorespiratory and Skeletal Muscle Physiotherapy, Universidade Federal de Juiz de Fora, Juiz de Fora, Brazil; ^2^Empresa Brasileira de Serviços Hospitalares/Hospital Universitário, Universidade Federal de Juiz de Fora, Juiz de Fora, Brazil; ^3^Department of Physiotherapy, Universidade Federal de Minas Gerais, Belo Horizonte, Brazil; ^4^Terapia Respiratória e do Sono, Pesquisa & Ensino, Belo Horizonte, Brazil

**Keywords:** community health, home care services, pulmonary rehabilitation, chronic obstructive pulmonary disease, telerehabilitation

## Abstract

This paper presents a rapid review of the literature for the components, benefits, barriers, and facilitators of pulmonary rehabilitation for chronic obstructive pulmonary disease (COPD) people in-home and community-based settings. seventy-six studies were included: 57 home-based pulmonary rehabilitation (HBPR) studies and 19 community-based pulmonary rehabilitation (CBPR) studies. The benefits of HBPR on exercise capacity and health-related quality of life were observed in one-group studies, studies comparing HBPR to usual care, and studies comparing to hospital-based pulmonary rehabilitation, although the benefits were less pronounced in the latter. HBPR reduced hospital admissions compared to usual care and was more cost-effective than hospital pulmonary rehabilitation. Most HBPRs were designed with low-density or customized equipment, are minimally supervised, and have a low intensity of training. Although the HBPR has flexibility and no travel burden, participants with severe disease, physical frailty, and complex comorbidities had barriers to complying with HBPR. The telerehabilitation program, a facilitator for HBPR, is feasible and safe. CBPR was offered in-person supervision, despite being limited to physical therapists in most studies. Benefits in exercise capacity were shown in almost all studies, but the improvement in health-related quality of life was controversial. Patients reported the benefits that facilities where they attended the CBPR including social support and the presence of an instructor. They also reported barriers, such as poor physical condition, transport difficulties, and family commitments. Despite the minimal infrastructure offered, HBPR and CBPR are feasible, safe, and provide clinical benefits to patients with COPD. Home and community settings are excellent opportunities to expand the offer of pulmonary rehabilitation programs, as long as they follow protocols that ensure quality and safety following current guidelines.

## Introduction

Pulmonary rehabilitation (PR) is the most effective non-pharmacological intervention for improving the physical and psychological condition of people with chronic obstructive pulmonary disease (COPD) (1, 2). Physical activity and exercise training are cornerstones of PR but challenging to embed in long-term disease management and everyday life. Despite the benefits of PR, adherence and completion rates remain low (3). Barriers to accessing PR are diverse, and the most common include geographic distance and difficulty with transportation, limitations caused by dyspnea symptoms and fatigue during commuting, difficulty reconciling work and/or domestic activities, and the limited availability of companion time (4). A survey study on content and the organizational aspects of PR programs, completed by representatives who had participated in the European Respiratory Society COPD Audit, encompassed 430 centers from 40 countries. This study demonstrated that most PR programs are offered in outpatient centers, followed by hospitals or both, and only 4.9% are offered elsewhere, such as at home or in community settings (5). Recent advances facilitate physical activity and exercise training in the home and community setting. An emerging study demonstrates the feasibility of physical training using minimal infrastructural resources in local contexts (domestic or community) (6). The rapid development of telehealth applications have assisted patients, families, and healthcare professionals and made PR affordable in the home environment (7). The use of technology in remote home monitoring and rehabilitation has increased due to convenience, innovation, customized services, and the scarcity of PR programs (8). Considering these issues, alternative means of providing rehabilitation, such as home-based (possibly telerehabilitation) or community rehabilitation, can increase access for and use by more patients.

This rapid review aims to investigate and discuss home and community-based interventions in the context of continuity of care and management of COPD, addressing the components, effects, barriers, and facilitators of PR in such home and community settings. With current challenges impacting the provision of continuity of care as PR for patients with COPD, a rapid review design was chosen to allow us to quickly investigate a large number of studies on PR at home and in the community as alternatives to center-based PR.

## Methods

The protocol was registered in PROSPERO on July 6, 2021.

### Types of Studies

Studies that reported physical exercise programs, interventions to increase physical activity, or PR at home and in community settings for people with COPD were included. Home-based pulmonary rehabilitation (HBPR) and community-based pulmonary rehabilitation (CBPR) were defined by their locations; in the participant's home and in a community-based setting (not in a hospital and not at home), respectively (6). Telerehabilitation is considered a form of remote supervision through the use of information and communication technologies (9), provided it is offered at home or in the community.

Cases studies were not included. Review articles were not included, but we reviewed their reference lists for other studies that met our inclusion criteria. There were no other restrictions on the study design. We included studies investigating the effects, barriers, and facilitators of physical activity, exercise training, behavior change, and self-management for COPD. Descriptive studies were included and in which the intervention was conducted at home or in community settings. Only studies published in English were included.

### Participants

We included studies in which participants were adults (18 years of age or older) and were diagnosed with COPD. We did not exclude studies based on sex, severity, or comorbidity.

### Search Methods for Identifying Studies

As this was a rapid review, we chose to search a single database, MEDLINE, for all publications dated up to April 6, 2021. We chose the MEDLINE database because of the availability of relevant MESH terms and good coverage of clinical topics in the English language. The search strategy for MEDLINE is presented in Supplementary Table 1. One author reviewed the titles and abstracts of the identified studies to determine their eligibility for inclusion.

### Data Extraction and Management

One author conducted data extraction using a standardized method and template, with random accuracy checks by a second author. The following information was extracted and categorized according to the setting (home or community):

- Methods of study (date/author, study design)
- Participants (age, disease severity, sample size)
- Intervention (physical exercise program components and co-interventions)
- Comparison
- Program supervision (in person, remote as web-based or phone calls, combined in person and remote, none)
- Duration
- Outcomes (primary outcome and other outcomes)
- Effects of intervention
- Facilitators
- Barriers

### Assessment of Risk of Bias

We considered the risk of bias according to the study design and the methods of analysis. As this was a rapid-review, we did not conduct a formal assessment using a risk of bias tool.

### Outcomes

Outcomes of interest were intervention components; supervision programs; effects on exercise capacity; physical activity; health-related quality of life (HRQoL); healthcare utilization, costs, and adverse events; and facilitators and barriers of rehabilitation programs implemented at home or in a community.

### Data Synthesis

A narrative synthesis was performed separately for each setting. We report feasibility, responsiveness to PR (e.g., post-rehabilitation changes), structured and supervised exercise training programs, an educational and behavioral program intended to foster long-term health-enhancing behaviors, and the provision of recommendations for exercise and physical activity, and barriers and facilitators for engaging in physical activity or exercise training in each setting.

## Results

### Home-Based Pulmonary Rehabilitation

**Figure 1:**
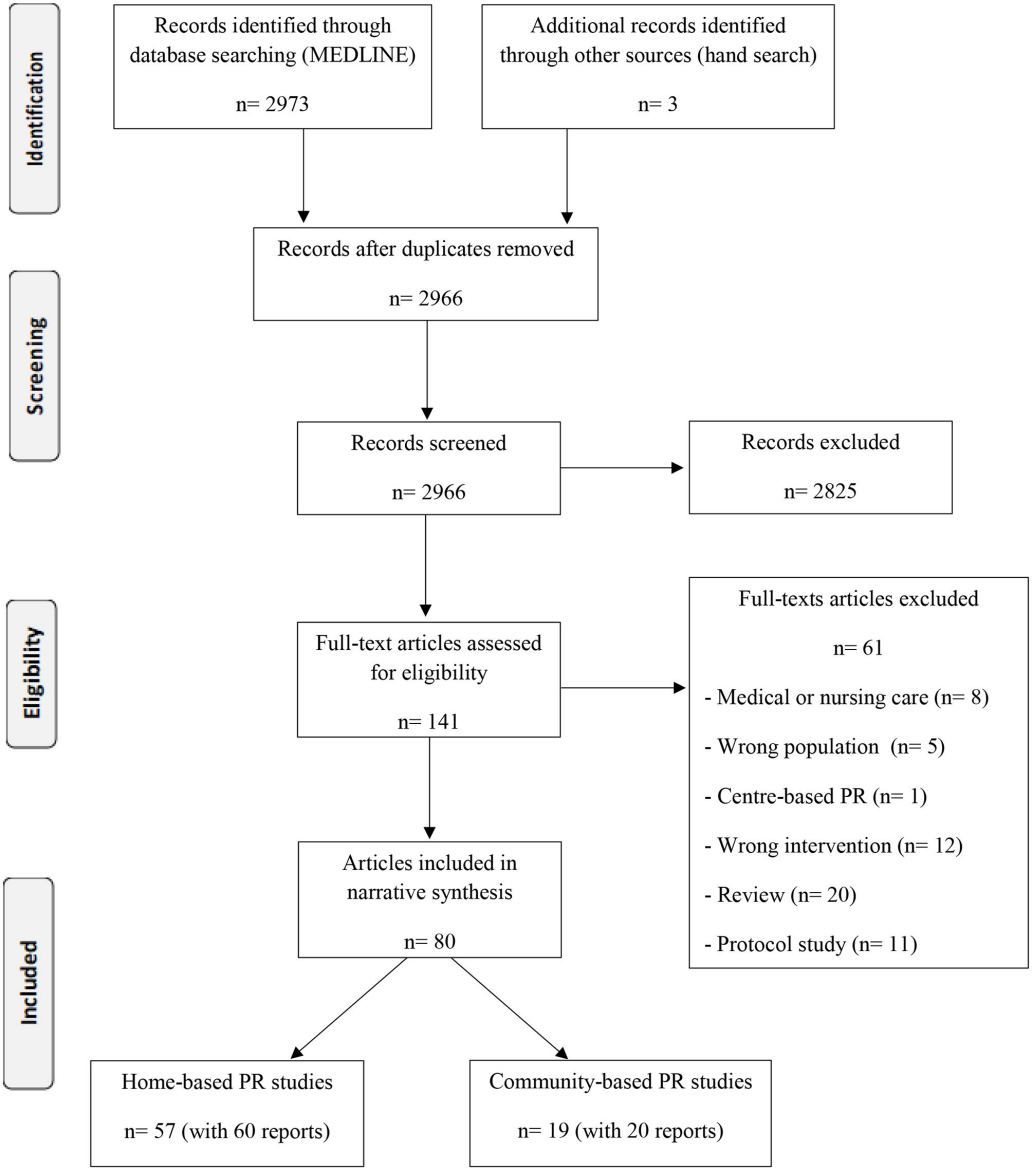
Flow diagram.

The characteristics of home PR studies are listed in Supplementary Table 2, including 57 studies (60 reports) (Figure 1): 39 randomized controlled trials (RCTs), 12 studies involving one-group pre-tests and post-tests, 3 qualitative studies, 3 non-RCTs.

#### Population

These studies enrolled people with a diagnosis of COPD, 40 years of age or older, presenting with forced expiratory volume in the first second of expiration (FEV_1_) ranging from 27.1 to 92% predicted, and most were clinically stable with no exacerbations during the previous 4–12 weeks. We identified four studies investigating participants' peri-exacerbations (9–14). Most studies involved participants with moderate disease severity, seven studies involved severe disease (13, 15–21), and one included mild disease (22).

#### Components of the HBPR Intervention

Twenty-nine studies added educational sessions to the exercise training program (13, 15, 17, 18, 20–45), and only six studies added psychological and/or dietary support (30, 34, 40, 46–48). The training strategy predominantly involved endurance exercises, sometimes combined with strength exercises, flexibility training, and, less frequently, stretching exercises.

Endurance exercises included different modalities and intensities, such as walking activity based on 125% of distance (10) and 80–90% of speed achieved in the six-minute walk test (6MWT) (17, 22, 25), walking to produce the dyspnea elicited at 75% of the distance from 6MWT (19), or walking based on a symptom-limited exercise test. Three studies prescribed walking intensity based on maximum oxygen consumption of 60–85% or maximum speed, both attained in the incremental shuttle walk test (ISWT) (32, 36, 46). In one study, participants performed speed walking following the music tempo on a mobile phone, set at 80% of the speed reached at ISWT (49). Another study prescribed a walking speed of 60% of the maximum VO_2_ obtained during an incremental exercise test (46). Two studies provided a portable metronome to maintain the prescribed walking pace (50, 51). A lower limb cycle ergometer was used with intensity prescription based on a heart rate of ~70% of maximum heart rate reached during the 1-min stepwise test (30) at 60% maximum work estimated from the initial 6MWT using an algorithm (31), or adjusted to meet the patient's capacity to endure for at least 30 min daily (52), based on perceived effort (39) or based on 60% of maximum load in the incremental ergometer test (21, 42, 44, 45). Other studies used walking, climbing stairs, cycling, and treadmill walking depending on the resources available in the patient's home or neighborhood (27, 40, 43, 53–55).

Strength training with anti-gravity exercises (20, 21, 44, 45, 56), sandbags, elastic bands, water bottles (22, 31, 46, 57, 58), free weights, or lower load dumbbells (11, 28, 30, 32, 33, 39, 48, 54, 59) were the most frequently used strategies. Functional exercises, such as step-up and step-down and sit-to-stand exercises, were also included in the regimen (22, 31). Five studies added inspiratory muscle training (17, 20, 21, 33, 42, 46). Two studies used flexibility and stretching exercises (34, 60). One study used a web-based exercise program on the web portal, which included breathing, relaxation, mobilization, resistance and endurance training, and mucus clearance exercises, in which the supervisor could freely select the exercises for each patient from the online exercise program (14).

Supervision was offered in person (10, 17, 20, 21, 23, 29, 33, 35, 36, 42, 44–46, 50, 58, 60–62), remotely by telephone or web (11, 12, 16, 19, 22, 24–26, 30–32, 34, 37, 40, 48, 49, 51, 52, 54, 56, 59, 63–67), or a combination of both (28, 39, 68).

The frequency of supervision was offered weekly (11–13, 20–27, 31, 34, 42, 44, 45, 48, 50, 52, 54, 56, 59–61), biweekly (9, 10, 29, 30, 35, 39, 46, 51, 53, 62, 64), progressing biweekly to monthly (17, 33), or every 4 weeks (32, 65).

Strategies for increasing motivation and adherence were evaluated using a daily manual diary (10, 15, 21, 22, 24, 25, 29, 31, 32, 36, 42, 50, 51, 61, 64, 69), a digital diary (15, 16, 48), pedometers (11, 33, 65), phone messages (15, 26), or through remote contacts (12, 14, 31, 34, 39, 43, 52, 59). One study reported moderate to high acceptability and validity of diary use compared to the accelerometer used during HBPR (70). HBPR studies showed a wide range of intervention durations ranging from 1 to 18 months.

#### Comparison

Twelve studies involved a pre- and post-intervention group without a comparison (11, 13, 15, 16, 19, 30, 36, 43, 47, 50, 56, 67). Most RCTs used a control group with usual care as standard treatment for COPD (10, 12, 14, 18, 20, 21, 23, 24, 26, 28, 39, 41, 44–46, 49, 54, 58–60, 62, 65, 69, 71, 72), which did not involve a supervised exercise training program. Nine studies (17, 22, 29, 33, 34, 51, 52, 57, 61) used attention control as a comparison in which participants received additional contact (e.g., a telephone call), but without supervised training. Seven RCTs (25, 31, 32, 37, 40, 41, 68) and one non-RCT (48) compared HBPR with hospital-based PR.

#### Outcomes and Effects

The main outcomes were exercise capacity, HRQoL, physical activity, utilization of health services, cost, and adverse events. Exercise capacity was assessed using the 6MWT in 34 studies (59%) (9, 10, 14, 17–20, 22–24, 27, 28, 31, 33, 35, 39, 42, 44, 46–49, 51, 54, 56–60, 65, 68, 69). Seven studies (13%) used the ISWT (25, 29, 32, 36, 37, 59, 62), three studies (5%) used the endurance shuttle walk test (ESWT) (25, 32, 59), eight studies (14%) used ergometer tests (20, 39, 42, 45, 54, 62, 68, 72), two studies (4%) used the time-up and go test (13, 58), two studies (4%) used the sit-to-stand test (13, 60), and one study (2%) used the 4-min walk test (41). Three studies (5%) (19, 36, 47) observed improvements in exercise capacity in a single group before and after HBPR. Twenty two studies (39%) reported an improvement in exercise capacity following HBPR compared to the control group (13, 17, 18, 23, 27, 29, 30, 33, 35, 41, 42, 44–46, 49, 51, 53, 54, 57, 59, 60, 62). Six studies (11%) indicated sustained benefits of exercise capacity after a maintenance HBPR program (9, 10, 39, 48, 65, 69). Four studies (7%) (31, 40, 41, 68) found equal improvements in exercise capacity in the comparison between hospital-based PR and HBPR. Two other studies (4%) (32, 37) revealed smaller improvements in exercise capacity for HBPR compared with hospital-based PR.

The health-related quality of life (HRQoL), using the Chronic Respiratory Questionnaire, was identified in 17 studies (29%) (9, 10, 21, 22, 25, 27, 31, 32, 37, 39, 45, 47, 57–59, 65, 68), 15 studies used the St. George's Respiratory Questionnaire (27%) (17, 19, 23, 35, 40, 43, 46, 48, 51, 53, 54, 56, 60, 68, 69), 5 studies (9%) used the COPD Assessment Test (CAT) (24, 25, 43, 48, 52), 4 studies (7%) used the short-form 36 (SF-36) (30, 36, 37, 56), 3 studies (5%) used the Clinical COPD Questionnaire (13, 14, 18), and 2 studies (4%) used EuroQol 5D (EQ-5D) (14, 43). Five studies (9%) (19, 30, 36, 47, 56) observed improvements in HRQoL in a single group after HBPR. Fourteen studies (24%) (17, 18, 21, 23, 28, 29, 33, 35, 45, 46, 52, 57, 60, 62) showed that HBPR increased HRQoL compared to the control group. Six studies (10%) (9, 10, 20, 48, 65, 69) reported that the improvement in HRQoL was preserved after the maintenance of HBPR compared to the control group. Five studies (9%) (31, 32, 37, 40, 68) observed similar improvements in HRQoL between HBPR and hospital-based PR.

Physical activity behavior was evaluated in nine studies (17%) (12, 26, 27, 33, 48, 51, 52, 59, 73). Five studies (9%) (26, 27, 33, 51, 52) observed improved physical activity behavior after HBPR compared to the control group. In three other studies (5%) (12, 59, 73), HBPR did not increase physical activity levels. One study (2%) (48) revealed that home-based maintenance was equally effective to hospital-based maintenance, outpatient, PR in preserving the initial improvement in time spent in sedentary, light, lifestyle and moderate daily physical activities over the 12-month period, and was superior to usual care exhibiting an increase in time spent in sedentary, and decrease in lifestyle, and moderate daily activities over 12 months of follow-up. Eight studies (15%) used diaries to reflect the level of adherence to exercise participation during unsupervised HBPR (14, 15, 36, 43, 51, 69, 70, 73). Two studies (4%) observed an increase in adherence to unsupervised short-term home exercise (69, 73).

Five studies (9%) assessed the utilization of health services (10, 23, 26, 43, 48). One study (2%) reported a reduction in the average length of stay (23). An RCT study (2%) observed a reduction in the number of long-term hospital admissions in the HBPR group compared to usual care (10). One study (2%) (26) observed a non-significant clinical reduction in hospitalization rates in HBPR. One pilot study, pre- and post-telerehabilitation intervention, suggested a reduction in healthcare utilization (43). One study (2%) reported that the hospital admission rate was equally effective between HBPR maintenance telerehabilitation and hospital-based PR (48).

Only three studies (5%) performed an economic analysis (13, 43, 74). Two of them applied home telerehabilitation. Rosenbek Minet et al. (13) observed that telehealth was more expensive than usual care but that it produced clinical benefits. Zanaboni et al. (43) identified a 27% reduction in hospital costs related to COPD with the use of telerehabilitation as a consequence of reduced access. Burge et al. (74) showed that HBPR was more cost-effective than hospital-based PR.

In general, details of adverse events were poorly reported. Ten studies (18%) reported no adverse events related to exercise training (27, 28, 31, 32, 35, 46, 51, 59, 68, 69). Only one (2%) study reported adverse events of mild muscle pain in the early stages of progressive resistance exercise, an episode of acute lower back pain, and a mild adductor strain that was resolved after a week of rest (58).

#### Facilitators of HBPR

HBPR facilitators were identified in two studies (22, 64). Two studies described HBPR as time-convenient and flexible, given that this program adapts to patients' routines, leading to decreased effects of interrupting daily activities and also reducing the travel burden by eliminating transport-related barriers (22). Additionally, patients reported that HBPR provided them with greater social support through frequent contact with the physiotherapist through one-on-one interactions and helped them achieve their personal goals (22). The training strategy used was customized according to the preference and availability of resources at home or in the neighborhood. Participants also reported that HBPR helped establish an exercise routine and improve self-management of the disease (22).

One study (64) identified the determinants of increased active behavior among people with COPD. Participants who exercised previously, with fewer depressive symptoms, and who lived with friends or family displayed higher walking frequency. The duration of walking was influenced by the level of physical conditioning. The consistency of walking over a year was determined by more supervised exercise sessions, as well as regular exercise before participating in the program, and the perceived benefits of social participation (64).

#### Barriers to HBPR

Five studies (9%) identified some barriers to rehabilitation in the home setting (11, 20, 61, 75, 76). Some of these barriers are fragmentation of the multidisciplinary team, difficulty in providing sources, addressing individual limitations in patients with pulmonary disease, and limited use of adjuvant actions (e.g., oxygen, non-invasive ventilation, neuromuscular electrical stimulation). In a qualitative study, patients reported that challenges included difficulties in initiating exercise in HBPR due to their prolonged sedentary lifestyle, some demotivation due to monotony in training due to the lack of exercise variety, and physical incapacity that impacts their ability to exercise (22). Some reasons for non-adherence to HBPR were identified as lack of motivation, anxiety, less in-person support, exacerbation, and comorbidity (77). Patients living alone and those who had not previously participated in primary PR program required additional support to continue and progress in the training program over an extended period of time. Other studies identified that the main reasons for declining HBPR after hospitalization for exacerbation of COPD were disinterest and significant illness or frailty (11). Patients who had severe disease and were less physically conditioned required support to maintain the duration of training at home to gain the benefits of exercise (64).

#### Telerehabilitation in the Home Setting

In 13 studies (24%), pulmonary telerehabilitation was used as an approach in the home environment for patients with COPD (11, 13–16, 24, 25, 27, 43, 48, 52, 59, 67). Ten studies (18%) used videoconference in real time (13–16, 24, 25, 27, 43, 59, 67). Some studies (5%) used asynchronous remote technology to transmit tele-monitored information such as vital signs, oxygen saturation, and symptoms (11, 48, 52). The use of telerehabilitation demonstrated good participant usability and acceptability, in addition to being safe and not triggering adverse effects related to the exercise intervention in eight studies (14%) (11, 13, 15, 16, 43, 48, 59, 67). Participants involved in telerehabilitation reported the proximity to their peers and therapists at home and the ability of the therapist to see the exercises being performed correctly as advantages (13). Telerehabilitation can be the solution to the fragmentation of the multidisciplinary team in the home environment (43). The participants showed very good compliance with the different components used in telerehabilitation. They reported ease of handling in monitoring and transmitting vital signs and oximetry data (48). Less elderly participants with better exercise capacity acquired autonomy in the components of lung telerehabilitation in fewer sessions than older participants and those with worse exercise capacity (78). Despite the expense generated by telerehabilitation compared to usual care, this approach brings clinical benefits to participants (13). Zanaboni et al. (43) reported that pulmonary telerehabilitation, due to reduced access to and short duration of health services, promoted a reduction of 27% in COPD-related costs. Barriers, such as dropout from telerehabilitation, were more frequently observed than for conventional hospital PR among participants with greater disease severity, lower exercise capacity, and a higher baseline anxiety level (25).

### Community-Based Pulmonary Rehabilitation

The characteristics of community-based PR studies are listed in Supplementary Table 3, including 19 studies (20 reports) (Figure 1): nine RCTs, one pilot study, five one-group pre- and post-test, three qualitative studies, and two non-RCTs. All studies included settings such as clubs, gyms, community centers, schools, or primary healthcare facilities for providing PR.

#### Population

The studies enrolled people with a diagnosis of COPD, who were 50 years or older, presenting with FEV_1_ predicted between 19.3 and 89.2%, and those who were mostly clinically stable without exacerbations during the previous 4–12 weeks, except one study that investigated participants during acute exacerbation (79).

#### Components of the CBPR Intervention

Thirteen studies (76%) added educational sessions to the exercise training program (75, 76, 79–89); only two studies added psychological and/or dietary support (79, 84). The training strategy predominantly involved endurance exercises and was most often combined with strength exercises and less frequently included flexibility and breathing exercises. The endurance exercises included different modalities and intensities. Walking activity was established based on ISWT results (81, 88, 90), based on symptoms of dyspnea rating, based on 60% or more of the maximum HR (89), perceived exertion between 4 and 6 on the modified Borg scale (79, 82, 91), walking on the treadmill at 75% speed achieved in the 6MWT (82), or based on the symptom-limited exercise test (88). Some studies used a strategy of combining several modalities of endurance training such as walking along a corridor, in a garden, or on a treadmill; cycling; or a climbing stairs (79–81, 86, 88–90, 92). These combined modalities defined exercise intensity based on the results of the symptom-limited exercise test (80, 81, 86, 88, 90, 92) or 60–80% of the estimated maximum heart rate (79). Two studies used endurance training or interval training as an option (81, 84). Some studies did not report the modality or intensity of the training and reported only that participants performed aerobic exercises (76, 85, 87, 93).

Strength training with exercises was offered using free weights (88, 92–94), machines (93), or elastic bands (92). Only three studies reported that intensity training was based on single repetition maximum (81, 82, 93). One study reported that strength training consisted of low-intensity circuit routines with weights (80). Five other studies reported only that participants performed strength training (79, 83, 84, 86, 87). Three studies added breathing exercises (79, 87, 89).

Supervision was offered in person in thirteen studies (76%) (75, 79, 81–84, 86–89, 92–94) and remotely by telephone calls in two studies (12%) (90, 91). One study initially offered in-person supervision and then remotely by phone calls (90) and another offered only phone calls (91). Although most of the sessions were supervised in community centers, some studies still used a diary (86, 91) or pedometers as strategies for increasing daily physical activity and adherence (81, 88, 90, 91). CBPR studies showed a wide range of duration from 3 weeks (pilot study) to 12 months.

#### Comparison

Seven studies did not have a comparison control group, four involved only a pre-and post-intervention group (76, 83, 84, 92), another two were qualitative studies (75, 87), and one pilot study (85). Six studies incorporated a control group with usual care in which participants received physical activity counseling (79, 82, 89–91, 93). Usual care did not involve a supervised exercise training program. Two studies used self-management educational sessions as a comparison, but without supervised training (81, 88, 94). Two studies (80, 86) compared CBPR with conventional hospital PR.

#### Outcomes and Effects

The main outcomes were exercise capacity, HRQoL, physical activity, utilization of health services, cost, and adverse events. Exercise capacity was assessed using 6MWT in seven studies (41%) (76, 80, 82, 83, 89, 91, 92), and seven studies (44%) used ISWT and ESWT (81, 83, 85, 86, 88, 90, 93). Three studies (19%) (76, 83, 92) observed improvements in exercise capacity in a single group before and after CBPR. Four studies (23%) reported an improvement in exercise capacity after CBPR compared with usual care (82, 89, 90, 93). Only one study (6%) (91) failed to detect improvement in exercise capacity compared to usual care, but this particular study offered an unsupervised walking program in predefined circuits to increase daily physical activity, not an exercise program for PR. Two studies (12%) (81, 86) observed improvements in exercise capacity when comparing CBPR and self-management intervention control groups. Two studies (12%) (80, 86) found an improvement in exercise capacity for both comparison groups between hospital-based PR and CBPR; however, in one study (6%) (86) the improvement in ESWT was greater in the hospital than in CBPR.

HRQoL was assessed in 15 studies (88%). The Chronic Respiratory Questionnaire was identified in seven studies (41%) (80–82, 85, 89, 91, 92), six studies (37%) used the St. George's Respiratory Questionnaire (76, 84, 86, 88, 90, 93), four studies (25%) (82, 86, 88, 91) used the Clinical COPD Questionnaire, one study (6%) (79) used the CAT and one study (6%) used the 15D questionnaire (83). Three studies (19%) (76, 83, 92) observed improvements in quality of life in a single group before and after CBPR. Three studies (19%) (82, 91, 93) did not detect an effect on HRQoL in the CBPR group compared to usual care. On the other hand, only one study (6%) (90) found an effect on HRQoL in the CBPR group compared to usual care. Two studies (12%) (81, 88) observed improvements only in the dyspnea domain in the CBPR group compared to the self-management group. Two studies (6%) (80, 86) reported similar improvements in HRQoL between the CBPR group and hospital-based PR.

Five studies (31%) evaluated physical activity behavior (81, 82, 88, 90, 91). Three studies (19%) (82, 90, 91) showed an improvement in daily physical activity in CBPR compared to usual care, which only provided counseling on physical activity. Two studies (12%) (81, 88) observed an improvement in physical activity behavior after a CBPR combined with the self-management intervention group compared to the self-management control group.

Health service utilization was evaluated in two studies (12%) (79, 84). In one study (6%) (79), none of the participants, either in the CBPR or control group, required hospitalization or used healthcare services. However, this was a pilot study with a short intervention of 3 weeks. Another study (84) observed a decrease in health service utilization over 1 year. One study (6%) (90) evaluated exacerbations and found no differences in the number of exacerbations between CBPR groups and usual care.

Only two studies (12%) performed economic analyses (84, 88). Golmohammadi's et al. (84) study identified the reduced direct costs associated with decreased health service utilization in a single pre- and post-CBPR group. Zwerink's et al. (88) study reported that CBPR cannot be considered cost-effective regarding exercise capacity compared to a self-management program after 2 years of follow-up, although the costs per patient with a relevant improvement in daily physical activity, and the cost per quality-adjusted life year were acceptable.

In general, details of adverse events were poorly reported. Five (31%) studies reported no adverse events related to exercise training (79, 81, 86, 91, 92). Only one (6%) study reported a higher frequency of lower extremity muscle pain during walks than patients in the usual care group (58).

#### Facilitators of CBPR

Three studies (16%) addressed facilitators for CBPR (84, 85, 95). Participants reported facilitators to be ease of access, perceived benefits, and convenient program components, beyond other factors, such as social participation (95). Other facilitators included reduced travel distances, easier parking, and a sense of community among the participants (85). The positive interactions and the supportive presence and flexibility of the exercise instructor contributed to participants' sense of safety and comfort within the program (75). Strategies such as including the participation of the next of kin in some CBPR sessions improved compliance (87).

#### Barriers to CBPR

Two studies (10%) reported barriers to CBPR (92, 95). A qualitative study (95) reported that perceived barriers to participating in CBPR included poor physical health, transportation difficulties, and family commitments. Another qualitative study identified barriers such as respiratory exacerbations, fatigue, barriers to access to transport, and weather (75). Patients in need of additional oxygen must carry their portable supply, which may limit their participation in community programs. Patients with unstable cardiovascular disease, uncontrolled diabetes, or complex comorbidities may be unable to safely perform exercises without access to immediate emergency response (96).

One study (6%) (96) listed some facility requirements to remove the barriers to CPRP: (i) a person trained in cardiopulmonary resuscitation on site, present at all times during sessions and able to call an ambulance in case of an emergency; (ii) bus routes to access the site; (iii) free (or low-cost) accessible parking; (iv) organized indoor or covered training area with floor space and with a minimum of 50 m lap distance; and (v) sanitary facilities <100 m from the training area (96). In the same study (96), a safety checklist was developed to reduce the probability of an adverse event and to prepare participants for emergencies. This checklist included: (i) following strict inclusion and exclusion criteria to carry out a CBPR; (ii) participants should be educated on location-specific emergency procedures and receive written instructions in case of an emergency; (iii) availability of face masks for cardiopulmonary resuscitation and first aid kits; (iv) participants should not be permitted to perform 6 MWT or physical training unless they bring their prescribed “rescue medication” (e.g., short-acting bronchodilator, glyceryl trinitrate); and (v) strict compliance with 6 MWT guidelines with the following modifications: continuous use of heart rate monitor and oximeter; impose rest if SpO_2_ <85%; discontinue testing if heart rate persistently >210–(0.65) age; exercise testing or training to start only if SpO_2_ >91% at rest and heart rate is 125 or 50 bpm; exercise testing or training ceased if the patient experiences chest pain, new arrhythmia, dizziness, or nausea.

## Discussion

This rapid review presented the components, intervention effects, barriers, and facilitators of PR programs in alternative settings, such as the home or community. The effects of HBPR on exercise capacity and HRQoL were observed in single-group studies, studies comparing HBPR to usual care, and studies comparing hospital-based PR, although the benefits were less pronounced in the latter setting. The results of the change in physical activity behavior were inconsistent, with some RCTs (26, 27, 33, 51, 52) reporting an increase in physical activity and others reporting no differences when compared to the usual care group (12, 59, 73). HBPR reduced the number of long-term hospital admissions compared to usual care in an RCT (26). HBPR using a telerehabilitation program in two non-RCT studies (43, 48) revealed a reduction in healthcare utilization. HBPR was more cost-effective than hospital-based PR in an RCT study (74). The discrepancies in results can be attributed to the heterogeneity of the programs in terms of duration, frequency of supervision, intensity of training, and fragmentation of the multidisciplinary team. In general, despite being cost-effective and individually tailored according to the needs of the participants, HBPR is minimally supervised, uses few resources, lacks a variety of exercises, and presents a low training intensity for patients. An HBPR designed with low-density or custom equipment can achieve similar effects to hospital-based PR as long as the prescribed parameters meet the guidelines of rehabilitation programs (31). Participants with severe disease, physical frailty, and complex comorbidities were the least adherent to HBPR (11), suggesting that this patient profile needs more support and supervision than that offered in hospital-based PR.

Studies using pulmonary telerehabilitation also covered the home setting. This included a variety of communications forms, such as synchronous interaction via videoconference or telephone calls and asynchronous interaction via text messages. Despite the costs associated with the implementation of technology, clinical benefits such as improved exercise capacity, reduced use of healthcare services, and good acceptability and safety were observed with telerehabilitation (13, 43). The monitoring and transmission of vital sign information, such as heart rate and oxygen saturation, were easily performed by the participants (48). Participants with severe disease, reduced exercise capacity, and higher levels of anxiety were those who had lower adherence to telerehabilitation (25). Participant involvement with technology and therapist support were key factors in participant autonomy (78). A recent systematic review showed that telerehabilitation programs promoted greater participant adherence compared to traditional PR (93 vs. 70% completion) (97). The same systematic review also reported that telerehabilitation produced results similar to traditional hospital-based in-person PR programs for exercise capacity, and compared to usual care (no rehabilitation) control, primary telerehabilitation trials can increase exercise capacity and can also help patients walk more (97). Telerehabilitation is a promising way to expand access for participants with barriers to traditional programs.

All CBPR models were safe and well-tolerated according to the included studies. Most CBPRs were performed with in-person supervision, with the exception of one program that aimed to increase physical activity through walks on previously established circuits without supervision. Benefits to exercise capacity were shown in all studies, except in a study involving walking in circuits, which was a different regimen than PR (91). The improvement in HRQoL was inconsistent between studies. Most RCT studies did not detect improvement compared to usual care, and two studies reported an improvement equivalent to hospital-based PR. An increase in active behavior was observed in the CBPR group compared to the self-management group and even compared to the usual care group. Few studies have analyzed healthcare utilization and CBPR costs, and those that did found no differences between groups, despite the incremental cost-effectiveness observed per patient with a clinical improvement in walking distance. None of the studies used telerehabilitation in a community setting. A long CBPR associated with self-management intervention was effective in achieving a behavioral change, which is reflected by an increase in daily physical activity after 1 year and maintained for the second year (88). CBPR can represent a form of transition between initial PR and a minimally supervised maintenance program (75). Although most CBPRs were conducted under the supervision of a physical therapist, a multidisciplinary primary healthcare team should be provided to ensure program consistency. Although there is evidence of clinical benefits, CBPR was heterogeneous and did not involve patients with severe comorbidities and those who required oxygen supplementation. The most frequent suggestions from participants about CBPR were to expand the number of program sites in the community and the need to subsidize the cost (85). Safety protocols and participant eligibility criteria for CBPR must be carefully established to avoid ignoring the risks of serious adverse events in non-specialized healthcare settings.

This rapid review has several limitations. We included only articles published in English and accessed them from a single electronic database (MEDLINE). Although we employed multiple broad search terms, we may potentially be missing relevant published information covered by other databases using other terms and in other languages. A single author performed the selection of studies and a single author performed data extraction with accuracy checks on a random sample by a second reviewer; using a standardized model for data extraction could perhaps have alleviated this limitation, which may have increased the risk of error and reduced confidence in the findings. We did not perform a formal methodological quality assessment. A formal risk of bias assessment may have identified important limitations to the conduct of the study and reporting that were not evident during this rapid review process. Therefore, the strengths of our conclusions may be reduced. The studies included a wide variety of components and protocols, which limited data synthesis but could be consistent for clinical application. Despite these limitations, the results shed light on important trends in the implementation of PR in home and community settings worldwide, including patient experience, facilities, and barriers reported in the studies.

In summary, endurance training was the physical intervention most offered, with walking being the most used modality. Co-intervention was infrequent and when offered, the most usual was educational sessions. There was a mix of in-person and remote supervision, with the frequency varying between weekly, biweekly, or every 4 weeks. HBPR and CBPR did not cause physical training-related adverse events for COPD patients. HBPR and CBPR were able to improve HRQoL and exercise capacity, but sometimes to a lower extent than hospital-based PR. HBPR provides the advantages of time convenience, flexibility, and reduced transportation challenges. CBPR is motivated by social support and the presence of an instructor. Barriers to both programs were related to poor physical health, increased symptoms, family commitments, and the community also included barriers to access transport. Telerehabilitation was an alternative feasible strategy, offering remote supervision in the home setting. Home settings and communities offer opportunities to expand the scope of PR programs. However, it is crucial to choose the optimal site according to the patient's preference and establish rehabilitation protocols that guarantee their quality and safety in accordance with existing guidelines.

## Author Contributions

TO: drafting of background and methods of protocol, data sifting, data extraction, and write-up of the full review. AP, GC, LS, and LA: data sifting, data extraction, revision of preliminary versions, and approval of the final version. MV: critical review of protocol, interpretation, drafting and revision of preliminary versions, and approval of the final version. CM: conceptual and clinical advice, drafting of background and methods of protocol, arbitrating conflicts, analysis and interpretation, and write-up of the full review. All authors contributed to the article and approved the submitted version.

## Funding

We are grateful for the support of the Aperfeiçoamento de Pessoal de Nível Superior—Brazil (CAPES)—Finance Code 001.

## Conflict of Interest

The authors declare that the research was conducted in the absence of any commercial or financial relationships that could be construed as a potential conflict of interest.

## Publisher's Note

All claims expressed in this article are solely those of the authors and do not necessarily represent those of their affiliated organizations, or those of the publisher, the editors and the reviewers. Any product that may be evaluated in this article, or claim that may be made by its manufacturer, is not guaranteed or endorsed by the publisher.
