# Supplementary material for: Embedding Pulmonary Rehabilitation for Chronic Obstructive Pulmonary Disease in the Home and Community Setting: A Rapid Review
Source: Front Rehabil Sci. 2022 Mar 30;3:780736. doi: 10.3389/fresc.2022.780736 (PMC9397727; doi:10.3389/fresc.2022.780736)
Supplement: Supplementary file 1 [file Table_1.pdf]

Embedding Pulmonary Rehabilitation in the Home and Community Setting – protocol for a mini review

Search methods for identification of studies:

The search strategy for Medline is shown below:

1. exp Lung Diseases, Obstructive/
2. exp Pulmonary Disease, Chronic Obstructive/
3. emphysema$.tw.
4. (chronic$ adj3 bronchiti$).tw.
5. (obstruct$ adj3 (pulmonary or lung$ or airway$ or airflow$ or bronch$ or respirat$)).tw.
6. (COPD).ti,ab.
7. 1 or 2 or 3 or 4 or 5 or 6
8. exp Physical Therapy Modalities/
9. exp Physical Fitness/
10. exp Physical Endurance/
11. exp Exercise Therapy/
12. exp Physical Exertion/
13. exp Exercise/
14. Respiratory Rehabilitation.tw.
15. Pulmonary Rehabilitation.tw.
16. exp Rehabilitation/
17. exp Respiratory Therapy/
18. Train*.tw.
19. Fitness*.tw.
20. Therap*.tw.
21. Interval train*.tw.
22. 8 or 9 or 10 or 11 or 12 or 13 or 14 or 15 or 16 or 17 or 18 or 19 or 20 or 21
23. exp Primary Health Care/
24. Primary Care.tw.
25. Community Care.tw.

26. Community-based.tw.

27. exp Community Health Services/

28. Home-based.tw.

29. exp Home Care Services/

30. Home rehabilitation.tw.

31. 23 or 24 or 25 or 26 or 27 or 28 or 29 or 30

32. 7 and 22 and 31

33. Limit 32 to English language
