# Supplementary material for: Embedding Pulmonary Rehabilitation for Chronic Obstructive Pulmonary Disease in the Home and Community Setting: A Rapid Review
Source: Front Rehabil Sci. 2022 Mar 30;3:780736. doi: 10.3389/fresc.2022.780736 (PMC9397727; doi:10.3389/fresc.2022.780736)
Supplement: Supplementary file 2 [file Table_2.docx]

**Supplemental Table 2.** Characteristics of included studies on home-based pulmonary rehabilitation

| **Study** | **Design** | **Age (Years)** | **FEV1 (%**  **predicted)** | **Sample**  **size** | **Home-based exercise**  **program** | **Comparison**  **intervention** | **Program supervision** | **Duration** | **Outcome** | **Effects** | **Barriers and facilitators** |
| --- | --- | --- | --- | --- | --- | --- | --- | --- | --- | --- | --- |
| Bauldoff 1996 (61) | RCT  Pilot study | HBG: 61 ± 14  CG: 63 ± 13 | HBG: 46 ± 20  CG: 55 ± 24 | 20 | Endurance: upper extremity exercises; the training level (weight used, sets, repetitions)  Co-intervention: none | Attention control | In-person once a week | 8 weeks | Upper- extremity endurance; hospital  admission | Increase endurance of unsupported arm exercise, reduce hospital admission | Not reported |
| Behnke 2000 (9) | RCT  Feasibility study | 68 ± 2 | 36 ± 7  Post- exacerbation patients | 30 | Practice walking at home (three times per day) at 125% of the best 6-min treadmill distance within 15 min  Co-intervention: none | Usual care | In-person, fortnightly during the first 3 months, and after maintained by monthly phone calls | 6 months | Exercise capacity: 6MWT and HRQoL: CRQ | Improvements in exercise capacity (6MWT) and HRQoL (CRQ) scores could be achieved after recovery from an exacerbation and these improvements were maintained after discharge, when supported by a home-based walking training | Not reported |
| Behnke 2003 (10) | RCT | HBG: 64 ± 1.9  CG: 68 ± 2.2 | HBG: 34.1 ±  7.4  CG: 37.5 ± 6.6 | 30 | Every day (three times/day) endurance training: walking for 15 min at 125% of 6-min treadmill distance  Co-intervention: none | Usual care | In-person, fortnightly during the first 3 months, and after maintained by monthly phone calls | 6 months | Exercise capacity: 6MWT and HRQoL: CRQ | The 6MTD and HRQL achieved in the hospital were fully maintained in the training group. Lower number of hospital admissions (total and disease-related) than control group | Not reported |
| Benzo 2018  (11) | Feasibility and qualitative study, telerehab. | At least 40 | Not specified | 15 | Endurance training: 12 min of slow walking Six full-body, low- intensity exercises (seated or standing exercises)  Co-intervention: none | None | Weekly phone calls. | 8 weeks | Adherence; Participant opinions about ease of system used | High overall adherence. | Barrier: Difficulty following video instructions and dealing with technology |
| Bernardi 2018 (50) | One-group pre- and post-test | 64 – 85 | Not specified | 21 | Endurance training: walk and step at home 30 min/day using a metronome 4 days/ week  Co-intervention: none |  | In-person, weekly for the first month to check that they maintained the correct step frequency | 12 weeks | Breathing pattern and oxygen saturation | Positive effects on the breathing patterns and reduced the perception of dyspnea | Not reported |
| Boxall 2005  (23) | RCT | HBG: 77.6 ± 7.6  CG: 75.8 ± 8.1 | HBG: 40.5 ±  5.9 | 46 | Endurance training:  low-intensity and | Usual care | Patients received an  average of 11 home | 6 months | Exercise  capacity: | Improvement in 6MWT, SGRQ scores,  and in the perceived breathlessness. | Not reported |

|  |  |  | CG: 37.7 ± 15 |  | symptom-limited walking exercise, 1 day/week. Walk levels progressed by increasing walk time (in minutes) Resistance training: upper limb strength training  Co-intervention: six educational sessions during the course of the  Program |  | visits during the course of the program |  | 6MWT; HRQoL: SGRQ;  Perceived breathlessnes s: Borg scale; Healthcare utilization; Length of stay at readmission | The intervention group had a significantly shorter average length of stay at readmission to hospital with exacerbation |  |
| --- | --- | --- | --- | --- | --- | --- | --- | --- | --- | --- | --- |
| Burkow 2013 (15) | Feasibility, Acceptabilit y study Telerehab. | At least 40 | Not specified | 5 | Every week patients participated in a group based exercise sessions by videoconference on TV  The home exercise program was based on the exercise program at the rehabilitation centre, and was intended to strengthen upper and lower extremities and to increase thorax flexibility  Co-intervention: health related educational sessions with multidisciplinairy team (e.g. pulmonary nurse, physiotherapist and  nutritionist) | None | Remotely by videoconference and phone calls | 8 weeks | Viability and acceptability of new system design | Good usability and acceptance | Not reported |
| Cameron- Tucker 2016 (24) | RCT  Telerehab | HBG: 68 ± 9.9  CG: 70 ± 6.8 | Not specified | 65 | Endurance training: walk at home 30 min/day everyday  Co-intervention: once- weekly education and self-management skills | Usual care | Two telefone calls weekly | 12 weeks | Exercise capacity: 6MWT; self- reported home-based walking; HRQoL: CAT  and health  behavior | There was no benefit to a telerehab on 6MWD, HRQoL and health behaviors compared to control group | Not reported |

| Chaplin 2017 (25) | RCT  feasibility, equivalenc e study Telerehab. | HBG:66.4 ± 10.1  CG: 66.1 ± 8.1 | HBG:58.7  ±29.1  CG: 55 ± 20.5 | 103 | Endurance training: walking at 85% of velocity of 6MWT and strength training  Co-intervention: motivational interviewing techniques and educational content of the web-based program | Conventional pulmonary rehabilitation | Patient’s progress was reviewed online and there was weekly contact via e-mail or telephone | 7 weeks | Feasibility (recruitment rates, eligibility, patient preference, dropout and completion rates); exercise capacity: ISWT and ESWT; HRQoL: CRQ  and CAT | There were no differences between the groups in any outcomes Dropout rates were higher in the web-based program (57% vs 23%)  The interactive web-based PR program was feasible and acceptable when compared with conventional PR. | Not reported |
| --- | --- | --- | --- | --- | --- | --- | --- | --- | --- | --- | --- |
| Cooke 2009  (56) | One-group longitudinal pre- and post-test Pilot study | 71.7 ± 8.45 | 41 (30 - 56) | 29 | A generic exercise program that included: a warm-up; strength exercises; balance exercises; and stretches  Co-intervention: none | None | Weekly telephone calls for the first month and at 2, 5, 8 and 10 months to assess and monitor progress | 12  months | Exercise capacity:6M WT; Self- efficacy (COPD self- efficacy scale); HRQoL: SF-36  and SGRQ | Most participants maintained: quality of life, exercise capacity; and self- efficacy over 6 months, with a slight decline in benefits 6 to 12 months after PR. | Not reported |
| Coultas 2018 (26) | RCT | HGB: 69.6 ± 9.4  CG: 67.3 ± 9.2 | HBG: 47.5 ±  12.3  CG: 47.3 ±  13.7 | 305 | Endurance training: 30 minutes of moderate intensity physical activity (e.g., walking, gardening, etc.) every day  Co-intervention: COPD self-management education and a behavioral intervention to promote lifestyle physical activity during a  6-week run-in period | Usual care | Weekly telephone calls | 20 weeks | Self-reported physical activity; health care utilization | There was an increase in self- reported physical activity, a reduction in sedentary activity and a decrease in the health care utilization | Not reported |

| De Roos 2018 (27) | RCT | 70.2 ± 9.5 | 67 ± 9.2 | 42 | Endurance training: Exercises sessions in centre-based consisted of 10 minutes of treadmill walking, 10 minutes of cycling and resistance exercises and unsupervised home walking program, with patients instructed to walk for at least 30 minutes  Co-intervention: education sessions for 5 minutes per week | Usual care | In-person weekly | 10 weeks | Daily PA was evaluated by accelerometr y and exercise capacity: 6MWT, HRQoL: CRQ  and exercise self-efficacy | Improved PA and exercise capacity in the HBPR group.  Adverse events: no adverse events were related to exercise training | Not reported |
| --- | --- | --- | --- | --- | --- | --- | --- | --- | --- | --- | --- |
| Dias 2013  (29) | RCT | HGB:66.5±5.8 CG: 64 ± 5.8 | HGB: 55.1 ±  24.8  CG: 60 ± 20.1 | 23 | Endurance training: walking for 40 minutes at 85% of peak oxygen consumption derived from ISWT  Co-intervention: respiratory exercises and education session on the first week | Active control, they received respiratory exercises and education sessions | In-person biweekly | 2 months | Exercise capacity: SWT;  Endurance of upper limbs; Respiratory muscle strength and endurance; HRQoL (AQ-  20) | There were no significant intergroup differences in the distance walked on the ISWT and HRQoL | Not reported |
| Dinesen  (16) 2013 | Qualitative study Telerehab | 67.9  (54.3 – 77.4) | None | 22 | A generic exercise program that included: sitting exercises on a chair, stretching of neck muscles, exercises for the legs, standing exercises for arms, exercises for chest cavity and walking exercises  Co-intervention: none | None | Once per month, remotely by telerehabilitation | 12 weeks | Patients’ attitudes towards telerehabilita tion | Indifference, learning as part of situations in everyday life, feeling of security and motivation for performing physical training | Not reported |
| Donesky 2011 (64) | RCT  Longitudina l | 66.3 ± 7.6 | 44.8 ± 14.3 | 103 | Endurance training: walking for at least 20 minutes three days/week  Co-intervention: none | None | Twice-monthly telephone calls | 12  months | Frequency (days per week), duration  (minutes per | Participants walked more frequently if they were exercising before they entered the study, had less depressive symptoms, and were living  with friends or family. Duration of | Barriers: Poor health was the major barrier to adherence; difficulty starting exercises |

|  |  |  |  |  |  |  |  |  | session), and continuity (persistence over 1 year) of home  walking | walks was influenced by supervised exercise training; living with spouse, friends, or family; and physical conditioning. The consistency of walking over a year was determined  by more supervised exercise sessions |  |
| --- | --- | --- | --- | --- | --- | --- | --- | --- | --- | --- | --- |
| du Moulin 2009 (65) | RCT | HBG: 67 (63 -  72)  CG: 72 (69 – 77) | None | 20 | Endurance training: quickly walk a distance equivalent to 125% of their last 6MWT with each training walk not exceeding 15 min, three times/day, every day  Co-intervention: none | Usual care | Telephone calls every 4 weeks | 6 months | Exercise capacity: 6MWT; HRQoL: CRQ | The maintaining home-based exercise training improved exercise capacity and HRQoL compared to control group | Not reported |
| Ferrari 2004 (30) | One-group longitudinal pre- and post-test | 70.4 ± 5.3 | 55.2 ± 17.5 | 28 | Endurance training: ergocycle for 35 to 40 minutes maintain heart rate around 70% of the maximum heart rate reached during the 1- min stepwise test. Three days/week  Resistance training for upper limbs  Co-intervention: education sessions for self-management of  COPD | None | Remotely, twice a month by phone calls | 12 weeks | 1-min stepwise exercise test; HRQoL: SF-36 | Improvement in quality of life and exercise tolerance | Not reported |
| Fernandez 2009 (17) | RCT | HBG: 66 ± 8  CG: 70 ± 5 | HBG:35.5 ± 11  CG: 38 ± 12 | 50 | Endurance training: walking, with time and velocity increasing progressively, until 30 minutes had passed at the 90% velocity reached in the 6MWT Resistance training for upper and lower limbs Respiratory reeducation and muscular inspiratory training. At least 5 days  per week | Active control with respiratory education sessions | In-person, twice monthly during the first 2 months of the program, and single monthly during the following 9 months | 12  months | Exercise capacity: 6MWT and HRQoL: SGRQ | Improvement in exercise capacity tolerance and HRQoL | Not reported |

|  |  |  |  |  | Co-intervention: respiratory education sessions |  |  |  |  |  |  |
| --- | --- | --- | --- | --- | --- | --- | --- | --- | --- | --- | --- |
| Franke 2016 (52) | Cross-over RCT | 63.7 ± 7.8 | 47.5 ± 15.8 | 53 | Patients received a bicycle ergometer with a personal introduction at home. The workload of the cycle training was adjusted to meet patients’ capability to endure for at least 30 minutes daily  Co-intervention: motivational phone calls | Active control | Remotely, by phone calls during intervention period | First and last 3 months | HRQoL: CAT  and self- reported physical activity | Improve regular physical activity and HRQoL | Not reported |
| Hernandez 2000 (62) | RCT | HBG: 64.3 ± 8.3  CG: 63.1 ± 6.9 | HBG: 41.7 ±  15.6  CG: 40 ± 16.4 | 60 | Endurance training: walking was performed at home or at a place near home (garden or a park), on a flat track that was 20 m long. The patient was supplied with a cassette that indicated the walking speed to him by means of an audible signal.  Training intensity was beginning at least 70% of the maximum speed attained in the ISWT  Co-intervention: none | Usual care | In-person every 2 weeks | 12 weeks | Exercise capacity: ISWT  (distance); Cycle ergometer test (VO2 max); Endurance test (time to submaximal); HRQoL: CRQ  (global, score); Dyspnea  (MRC) | There were not improvements in maximal effort parameters in the ISWT and in the cycle ergometer, but with improvement at time walking and distance in the submaximal exercise test, and dyspnea and HRQoL | Not reported |
| Holland 2013 (67) | One-group longitudinal pre- and post-test Feasibility study, telerehab | 66 (56 – 83) | 66 ± 18 | 8 | Endurance training: cycling for 30 min at 60% of peak work estimated from the initial 6MWT using a algorithm for cycle exercise. Two days/week  Co-intervention: education sessions for self-management of  COPD | None | Remotely supervised the exercise training sessions for two participants at a time | 8 weeks | Adverse events; sessions attended; system usability; exercise capacity: 6MWT; HRQoL: CRQ | Real time telerehabilitation was safe and feasible | Not reported |

| Holland 2017 (31) | RCT  equivalenc e study | HBG: 69 ± 13  CG: 69 ± 10 | IG: 52 ± 19  CG: 49 ± 19 | 166 | Endurance training: at least 30 min using a modality accessible, which was usually walking. Participants recorded the distance walked using a pedometer. Resistance training: functional activities and equipment that were accessible in the home environment, including sit-to-stand from a dining chair, step ups on an internal or external step, and water bottles for upper limb weights  Co-intervention: Self- management training included structured (lecture-based) and unstructured disease management education and goal setting | Centre-based pulmonary rehabilitation  : 30 min of aerobic training (walk training) and cycle training Resistance exercises: stair climbing and sit-to- stand practice, as well as free weights for the upper limbs | Weekly phone calls | 8 weeks | Exercise capacity: 6MWT; HRQoL: CRQ | Home-based pulmonary rehabilitation program provides improvements in functional exercise capacity and HRQoL that was equivalent to conventional centre- based pulmonary rehabilitation.  Adverse events: no adverse events were related to exercise training | Not reported |
| --- | --- | --- | --- | --- | --- | --- | --- | --- | --- | --- | --- |
| Hornikx 2015 (12) | RCT  Pilot study | HBG: 66 ± 7  CG: 68 ± 6 | HBG: 38 ± 17  CG: 48 ± 18  After an exacerbation of COPD | 30 | Physical activity with real-time feedback using a pedometer based on step counts and counseling by telephone calls to motivate patients to increase their PA level during 1 month  Co-intervention: none | Usual care | Remotely, three times a week telephone calls | 1 month | Physical activity level | Real-time feedback and physical activity counseling did not result in better improvements in comparison to usual care in patients post exacerbation of COPD | Not reported |
| Horton 2017 (32) | RCT  Equivalenc e study | HBG: 68 ± 9  CG: 67 ± 8 | HBG: 47.8 ±  18.6  CG: 48.7 ±  17.1 | 287 | Endurance training: daily walks at least 30 minutes with walking speed prescribed at 85% | Conventional centre-based PR program consisted of  twice weekly, | Telephone calls during the 7 weeks | 6 months | Dyspnea: CRQ and exercise capacity: ISWT and  ESWT | The home-based program achieved improvements in dyspnoea and exercise endurance capacity to a similar level to conventional  supervised PR | Not reported |

|  |  |  |  |  | of predicted VO2 peak from the ISWT Resistance training program  Co-intervention: educational sessions for both groups | involving exercise and education. During one of the supervised sessions per week participants completed some resistance exercise |  |  |  | Adverse events: no adverse events were related to exercise training |  |
| --- | --- | --- | --- | --- | --- | --- | --- | --- | --- | --- | --- |
| Kawagoshi 2015 (33) | RCT | HBG: 74.5 ± 8.5  CG: 75 ± 9 | HBG: 59.3 ±  22  CG: 60.6 ±  20.8 | 27 | Endurance training: walking for at least 15 min with feedback using a pedometer  Resistance training for upper and lower limbs, including sitting and calisthenics, inspiratory muscle exercises  Co-intervention: education program monthly | Active control involving the same program with no pedometer feedback | In-person, every two weeks | 12  months | Physical activity in daily life, exercise capacity: 6MWT,  respiratory and quadriceps muscle force, functional status, and HRQoL: CRQ  scores | Home-based program, with the feedback from using pedometer was effective in improving daily physical activity, inspiratory strength, 6MWD, and HRQoL (CRQ scores) | Not reported |
| Khoshkesht 2015 (34) | RCT | 56.6 ± 8.8 | None | 66 | Breathing exercises and muscle stretching exercises, warming exercise, and cooling  Co-intervention: introductory education sessions, diet therapy, stress reduction methods, respiratory  Exercises | Active control: involving a program based on the Bandura's self-efficacy theory. | Weekly phone calls | 7 weeks | Self-efficacy | The home-based pulmonary rehabilitation improved the self- efficacy | Not reported |
| Lahham 2018 (22) | Qualitative study | 66.3 | 56.1 | 13 | Endurance training: at least 30 min using a modality accessible to the participant, which  was usually walking. | Centre-based pulmonary rehabilitation involved at  least 30 min | Weekly phone calls | 8 weeks | Patient perspective (challenges and positive  impact of | Barriers: difficulties in initiating exercise, lack of variety in training and physical incapability  Facilities: flexibility and convenience  of the program; social support | Barriers: difficulties in initiating exercise, lack of variety in training and physical incapability |

|  |  |  |  |  | Participants recorded the distance walked using a pedometer. Resistance training: functional activities and equipment that were accessible in the home environment, including sit-to-stand from a dining chair, step ups on an internal or external  Co-intervention: Self- management training included structured (lecture-based) and unstructured disease management education and goal setting | of aerobic training was performed each session, including walking training (treadmill or corridor) and cycle training. Resistance exercises used functional activities |  |  | home-based pulmonary rehabilitation  ) | received, both from the physiotherapist over the phone and from family and friends who encouraged their participation; Positive impact: HBPR had helped establish an exercise routine and improved their disease management, physical fitness and symptom reduction | Facilities: flexibility and convenience of the program; social support received, both from the physiotherapist over the phone and from family and friends who encouraged their participation; HBPR had helped establish an exercise routine and improved their disease management; positive impacts on physical fitness and symptoms |
| --- | --- | --- | --- | --- | --- | --- | --- | --- | --- | --- | --- |
| Lahham 2020 (73) | RCT | HBG: 68 ± 9  CG: 67 ± 10 | HBG: 90 ± 8  CG: 92 ± 7 | 58 | Endurance training: walking speed was set at 80% of the speed walked during a 6-minute walk test (6MWT). Resistance training comprised upper and lower limb exercises using equipment available at home (home stairs for step ups and sealed water bottles as weights)  Co-intervention: none | Active control: participants received eight once- weekly social phone calls to control for attention | Weekly phone calls | 8 weeks | Exercise capacity: 6MWT, HRQoL: CRQ  and PA level | Both groups showed improvements in exercise capacity, symptoms and HRQoL over time, however, there was no difference in 6MWD at end- intervention | Not reported |
| Lee 2013  (35) | One-group longitudinal pre- and post-test | 66.2 ± 7.8 | 48.7 ± 16.5 | 27 | Endurance training: walking speed at 60% of maximum work rate of 6MWT  Resistance muscle training and respiratory muscle training for 12  weeks | None | In-person every two weeks | 12 weeks of intervent ion; 6 months of follow- up | Exercise capacity: 6MWT; HRQoL: SGRQ | Improve the exercise capacity and quality of life over the six-month follow-up period after rehabilitation Adverse events: no adverse events were related to exercise training | Not reported |

|  |  |  |  |  | Co-intervention: education sessions and breathing exercises |  |  |  |  |  |  |
| --- | --- | --- | --- | --- | --- | --- | --- | --- | --- | --- | --- |
| Li 2020  (75) | One-group longitudinal pre- and post-test | 65.1 ± 8.5 | 48.9 ± 12.4 | 418 | Endurance training: outside walking, 3 times per week. The targeted HR would be suggested to 75% of the maximum HR, starting with 5 minutes and progressively increasing to 20 minutes Resistance muscle training and respiratory muscle training once every day for 2 months  Co-intervention:  education sessions | None | Once-a-week phone call interview and self- report diary supervise in two months | 8 weeks | Exercise capacity (6MWT);  Smoking consumption; Exacerbation; Smoking status; Pharmacologi cal therapy | 170 patients (40.7%) who completed less than 50% sessions of the PR program were categorized as  “nonadherence.” Compared to  completers, “nonadherence” patients had more cigarette consumption, higher emotional score, less 6MWD, more exacerbation, using nebulizer frequently, and higher rate of smoking at enrollment | Main barriers: exacerbation; patients considered themselves to be too ill to complete the program or regarded the pulmonary rehabilitation as so difficult task to perform even post-exacerbation; this phenomenon may be so-called “lack of interest or motivation”. |
| Liu 2019  (60) | RCT | 65.3 ± 9.0 | The most patients had GOLD stage between II – III | 37 | Warm-up exercise for 10 min, focusing on dynamic flexibility exercises of involved muscle groups and stretching muscles and pulmonary exercises  Co-intervention: none | Usual care | In-person weekly | 3 months | Exercise capacity: 6MWD, 30s  arm curl test, 30s sit-to- stand test, HRQoL: SGRQ) | There was an improvement in the exercise capacity of the upper and lower limbs, and endurance capacity | Not reported |
| Maltais 2008 (68) | RCT  Equivalenc e study | HBG: 66 ± 9  CG: 66 ± 9 | HBG: 46 ± 13  CG: 43 ± 13 | 252 | Endurance training: cycling for 40 minutes at 60% maximum work rate achieved during a test of peak exercise capacity  Resistance training  Co-intervention: none | Conventional outpatient hospital- based exercise program | Weekly phone calls | 8 weeks | HRQoL: CRQ  and SGRQ); Exercise capacity: incremental cycle exercise test and 6MWT;  adverse event | Both interventions produced similar improvements in CRQ-dyspnea subscale, health status in SGRQ (at 1 year), 6MWD and cycling endurance time (at 3 months)  Adverse events: no adverse events were related to exercise training | Not reported |
| Minet 2015  (13) | One-group longitudinal pre- and post-test, telerehab, | 69.2 ± 8.8 | 27.1 ± 12.5 | 50 | Endurance training with an intensity of 60%-90% of max capacity, strength training with an intensity of 60% of 1 | None | Supervised exercise sessions by videoconferencing | 3 weeks | Health status: Clinical COPD Questionnair e and physical performance:  sit-to-stand | Improvements in health status and in the physical performance | Not reported |

|  | feasibility study |  |  |  | Repetitions Maximum and breathing exercises  Co-intervention: energy conservation techniques by an occupational therapist, patient education |  |  |  | test and a timed-up- and-go test |  |  |
| --- | --- | --- | --- | --- | --- | --- | --- | --- | --- | --- | --- |
| Nasciment o 2015 (36) | One-group longitudinal pre- and post-test | 64.8 ± 5.1 | 55.7 ± 20.7 | 14 | Endurance training: walking for 40 minutes at 85% of maximum speed achieved in ISWT and based Borg dyspnea scale  Resistance training  Co-intervention: one  education session after baseline assessment | None | Visits were scheduled to laboratory, during which patients demonstrated their physical exercise routine from the HBPR program | 8 weeks | Dyspnea (MRC); HRQoL (SF-  36); Lung function; Respiratory muscle strength; Incremental test for upper limbs; ISWT;  IL-6 and IL-8 | Increase in the walked distance, the maximal inspiratory pressure; improvements on two components from the HRQoL, and a decrease in plasma IL-8 levels after the intervention | Not reported |
| Nolan 2019  (37) | Non-RCT:  propensity- matched cohort study | HBG: 71 ± 10  CG: 71 ± 9 | HBG: 45.3 ±  20.3  CG: 45.7 ±  19.7 | 308 | Endurance training: exercise to achieve a Borg dyspnea scale score of 3–4 and progression included increasing the time to achieve 30 min Resistance training for upper and lower limbs with home-made weights as water bottles  Co-intervention: educational material | Outpatient program according to the  British Thoracic Society Quality Standards. Two supervised sessions per week  (aerobic and resistance training) and at least one unsupervised home exercise  session was | Weekly telephone follow-up | 8 week | Exercise capacity: ISWT and HRQoL: CRQ | Smaller improvements in exercise capacity with home-based exercise compared with PR, but similar improvements in quality of life | Not reported |

|  |  |  |  |  |  | encouraged per week. |  |  |  |  |  |
| --- | --- | --- | --- | --- | --- | --- | --- | --- | --- | --- | --- |
| Oh 2003  (38) | RCT | HBG: 64.8 ± 7.8  CG: 66.8 ± 12.2 | HBG: 42.1 ±  15  CG: 44.9 ±  17.7 | 23 | Endurance training: walking, stair climbing, and stepping.  Stretching exercise with theraband.  Inspiratory muscle training  Co-intervention: education, psychosocial components that include relaxation and telephone calls | Active control: educational advice | Twice a week phone calls | 8 weeks | Exertional dyspnea Borg, exercise capacity: 6MWD, and HRQoL: CRQ | The experimental group showed a lower level of exertional dyspnea, more exercise tolerance, and greater improvement in health-related quality of life than the control group | Not reported |
| Na 2005  (46) | RCT | HBG: 63.8 ±  10.3  CG: 64.4 ± 5.4 | HBG: 41.3 ±  11.4  CG: 39.7 ±  12.8 | 43 | Endurance training by walking (started from 60% of VO2max Resistance training with elastic bands Inspiratory muscle training for 15 min day  Co-intervention: education, psychosocial and nutritional support | Usual care | Visited hospital every two weeks for evaluation and a new exercise regimen | 12 weeks intervent ion, 15 months follow-up | Exercise capacity: 6MWT and HRQoL: SGRQ | In the experimental group, quality-of- life, VO2 max and Wmax were significantly improved compared to the control group. Lower extremity endurance was well maintained up to one year after the completion of the training  Adverse events: no adverse events were related to exercise training | Not reported |
| O’Shea  2007 (58) | RCT | HBG: 66.9 ± 7.0  CG:68.4 ± 9.9 | HBG 49 ± 25  CG: 52 ± 22 | 54 | Resistance training consisting of six progressive exercises of hip abduction in standing, simulated lifting, sit-to-stand, seated row, lunges,  and chest press three sets of 8 to 12 repetitions against elasticized resistance bands  Co-intervention: none | Usual care | In-person weekly, and phone calls at 6, 12,  18, and 24 weeks of the trial | 12 weeks intervent ion, 12 weeks follow-up | Strength muscle and walking capacity, HRQoL (CRQ),  exercise capacity: 6MWT,  Timed Up and Go, Upper limb activity was measured using the Grocery  Shelving Test | There was an improvement in knee extensor strength, but it did not lead to improvements in activity and participation  Adverse events: mild muscle pain in the early stages of resistance exercise, an episode of acute low back pain and mild adductor strain | Not reported |

|  |  |  |  |  |  |  |  |  |  |  |  |
| --- | --- | --- | --- | --- | --- | --- | --- | --- | --- | --- | --- |
| Pande 2005  (47) | One group Prospective pilot study | Not specified | 39.7 ± 15.3 | 24 | Leg exercise training for 20 min every day.  Breathing exercises: incentive spirometer 20 times every day and diaphragmatic breathing and pursed lip breathing for 10 min every day  Co-intervention: 30- minute interactive education session focusing on causes, manifestations and management of COPD. Inhaled medication, nutritional support, psychosocial support | None | Not specified | 6 weeks | Maximal mid- expiratory flow rate, dyspnoea assessment using  visual analogue scale, exercise capacity: 6MWT,  maximum inspiratory pressure and HRQoL: CRDQ | There was improve exercise endurance, dyspnea sensation, and quality of life. | Not reported |
| Pinto 2014  (28) | Brief report | HBG: 68.9 ± 9.2  CG: 71.9 ± 7.6 | HBG: 33.5 ±  7.3  CG: 34.5 ± 9.5 | 41 | Endurance training: Walking, stair climbing, cycling, and treadmill walking. Initially, training duration was 20-30 minutes and then increased to 1 hour.  The training intensity was self-limited by dyspnea and leg fatigue (moderate to relatively severe on the Borg scale)  Breathing and stretching exercises and strength exercises for upper and lower limbs  Co-intervention: education and self- management in the first two sessions with  physiotherapist | Usual care | Two weekly visits by the physiotherapist in the first 2 weeks, with follow-up visits twice a month and weekly telephone calls | 12 weeks | Exercise capacity (6MWT);  Dyspnea (LCADL); HRQoL (SGRQ) | There were statistically significant differences between groups in the total score of SGRQ. The HBG demonstrated statistically significant differences in all domains of the London Chest Activity of Daily Living Scale and in 6-minute walk distance Adverse events: no adverse events were related to exercise training | Not reported |

| Pradella 2005 (54) | RCT | HBG: 62.4 ±  10.7  CG: 65.3 ± 8 | HBG: 43.9 ±  16.2  CG: 54.0 ±  22.2 | 44 | Endurance training: 40  min walking 60 –70% of the maximum heart rate. The stair exercise began with 5 min/d, increasing to 15 min after 2 weeks. If a subject did not have stairs at home, a platform 20 cm in height and 50 cm in depth.  Upper limb exercises of diagonal movements with a 1-kg load  Co-intervention: educational booklet, a log to record their activities | Usual care | Weekly phone call | 8 weeks | Exercise capacity: 6MWT and endurance test; and HRQoL: SGRQ | There was improve in exercise capacity and quality of life | Not reported |
| --- | --- | --- | --- | --- | --- | --- | --- | --- | --- | --- | --- |
| Pomidori 2012 (51) | RCT | HBG: 70 ± 9  CG: 74 ± 7 | HBG: 48 ± 13  CG: 49 ± 12 | 47 | Endurance training: Speed walking paced by a metronome, the mean speed was recorded during the 20 minutes of SWT and was chosen as the walking speed, with a fixed distance for 20 to 30 minutes per day  Co-intervention: none | Active control: they walked a known distance in a given period of time, with a fixed distance (walking path located near home) without paced by a  metronome | In-person  weekly for the first month and twice a month by phone calls | 12  months | Exercise capacity: 6MWT, and PA: armband, and HRQoL: SGRQ | All subjects showed a significant improvement in the 6MWT after 1 year, but the improvement was higher in interventional group: 6MWT, mMETs daily average, best in the intervention group  Adverse events: no adverse events were related to exercise training | Not reported |
| Ries 2003  (39) | RCT | 67.1 ± 8.2 | 45 | 155 | Endurance training: walking exercise was begun on a treadmill and subsequently translated into an appropriate walking pace for home training. Duration up to 20 to 30  minutes of continuous | Usual care | Weekly phone calls and in-person monthly | 12  months of home- based maintena nce after 8 weeks of | Exercise capacity: 6MWT,  maximum treadmill with expired gases:  VO2max, and HRQoL: CRQ | During the 12-month intervention, exercise capacity (maximum treadmill workload and 6MWT and overall health status ratings were better maintained in the intervention group. There were no group differences for self-efficacy, generic and disease- specific quality of life, and health care  use | Not reported |

|  |  |  |  |  | walking with increase in level as tolerated by symptom-limits Resistance training: upper extremity training of arm lifts developed for pulmonary patients  Co-intervention: education sessions |  |  | conventi onal PRP | total, health care use |  |  |
| --- | --- | --- | --- | --- | --- | --- | --- | --- | --- | --- | --- |
| Sindhwani 2011 (18) | Non-RCT  Pilot study | HBG: 60.5 ± 4.6  CG: 61.3 ± 5.9 | HBG: 43.6 ±  2.9  CG: 43.8 ± 3.0 | 20 | Endurance training and respiratory training: diaphragmatic breathing, pursed lip breathing and  chest expansion exercise  Co-intervention: educational session regarding COPD using audiovisual aids and  dietary instructions | Usual care | In-person, monthly | 6 months | Exercise capacity: 6MWT and HRQoL:  clinical COPD questionnaire | Improves the quality of life and exercise endurance | Not reported |
| Spencer 2010 (69) | RCT | HBG: 65 ± 8  CG: 67 ± 7 | HBG: 57 ± 21  CG: 60 ± 16 | 48 | Supervised conventional program plus maintenance unsupervised home exercise program Endurance training: 20 min walking (track or treadmill), 20 min cycling, 10 min arm cycling  Resistance training: upper and lower limb strength exercises  Co-intervention: none | Active control: they received the exercise booklet and diary | Supervision only for the first session in the gymnasium | 12  months | Exercise capacity: 6MWT; HRQL: SGRQ | Exercise capacity and quality of life were successfully maintained at the 12-month follow-up  Adverse events: no adverse events were related to exercise training | Not reported |
| Stickland 2011 (40) | Non-RCT  Equivalenc e study Telerehab. | HBG: 69.2 ± 8.6  CG: 69.5 ± 9.7 | HBG: 48.3 ±  24.9  CG: 48.9 ±  19.2 | 409 | Endurance training: walking on track or treadmill,  cycling and arm ergometer training Resistance training:  upper and lower limb. | Conventional outpatient hospital- Based exercise program | Weekly, via telerehabilitation | 6 months | HRQoL: SGRQ,  exercise capacity: 12MWT | There was an improvement in quality of life and exercise capacity comparable to the PR-standard | Not reported |

|  |  |  |  |  | Flexibility exercises and breathing retraining via telehealth  Co-intervention: education sessions via telehealth |  |  |  |  |  |  |
| --- | --- | --- | --- | --- | --- | --- | --- | --- | --- | --- | --- |
| Strijbos 1996a;  1990 (55,  72) (two reports from the same study) | RCT | HBG: 59.9 ± 7.8  CG: Not available (no significant differences between groups- reported) | HBG: 50.2 ±  12.7  CG: Not available  (no significant differences between groups- reported) | 30 | Endurance training: different types of exercises were used (e.g., cycling, walking, stairclimbing, and training of upper extremities); cycling at 60%-75% Wmáx; relaxing exercises and breathing exercises, 2 days/week  Co-intervention: none | Usual care | In-person, weekly | Not available | Exercise capacity: incremental symptom- limited cycle test | Improved the exercise tolerance | Not reported |
| Strijbos 1996b (41) | RCT  Equivalenc e study | HBG: 60.0 ± 7.8  CG1: 61.2 ± 5.5  CG2: 63.1 ± 5.1 | HBG: 82.8 ±  20.6  CG 1: 79.5 ±  20.4  CG2: 83.5 ±  16.2 | 45 | Endurance training: exercise reconditioning walking and stair climbing up to 70% of the maximum workload Relaxing exercises and breathing exercises  Co-intervention: educational session | CG1: hospital based outpatient PR, CG2:  Usual care | In-person, | 18  months | Exercise capacity: 4MWT and incremental symptom- Limited cycle ergometer test | Similar improvements were detected in exercise capacity and in Borg dyspnea and leg effort scores at similar work levels during the cycle test | Not reported |
| Tabak 2014  (14) | RCT  Pilot study telerehab. | HBG: 64.1 ± 9.0  CG: 62.8 ± 7.4 | HBG: 50.0  [33.3–61.5]  CG: 36.0  [26.0–53.5] | 29 | Web-based exercise program with a standardized exercise protocol: physiotherapist could freely select the exercises for each patient for the online exercise program  Co-intervention: technology-supported care program: self- management, changing  activity behavior, and | Usual care | Remotely, via web portal and activity coach smartphone | 9 months | Exercise capacity: 6MWT,  fatigue: Multidimensi onal Fatigue Inventory 20, HRQoL:  Clinical COPD Questionnair e, EuroQoL- 5D  Adherence to  the online | The web portal was used in 86.4% of days, web exercise adherence 21.0%, satisfaction with received care maximum score. Number of hospitalizations, emergency room variables descriptive only. Only the MRC was significantly different between the groups | Not reported |

|  |  |  |  |  | telemedicine, a web- based exercise program, breathing exercises, relaxation, mobilization, text description and movie |  |  |  | diary, adherence to the exercise, health care utilization |  |  |
| --- | --- | --- | --- | --- | --- | --- | --- | --- | --- | --- | --- |
| Tsai 2016  (59) | RCT  Telerehab. | HBG: 73 ± 8  CG: 75 ± 9 | HBG: 60 ± 23  CG: 68 ± 19 | 37 | During an initial home- visit prior to training, a laptop computer with an in-built camera a stationary lower limb cycle ergometer and a finger-tip pulse oximeter were delivered to the participant’s home Endurance training: lower limb cycle ergometry, walking training  Resistance training exercises  Co-intervention: none | Usual care | Remotely supervised in real-time by videoconferencing | 8 weeks | Exercise capacity: 6MWT, ISWT,  ESWT; self- efficacy; HRQoL: CRQ  and physical activity | There was an increase in endurance shuttle walk test time and in self- efficacy when compared to usual care, with no difference for other outcomes  Adverse events: no adverse events were related to exercise training | Not reported |
| Vasilopoulo 2017 (48) | RCT  Telerehab. | HBG: 66.9 ± 9.6,  CG1: 66.7 ± 7.3  CG2: 64.0 ± 8.0 | HBG: 49.6 ±  21.9  CG1: 51.8 ±  17.3  CG2: 51.7 ±  21.0 | 147 | After 2-month initial PR program, the home- based maintenance tele- rehabilitation program was performed comprised arm and leg exercises, as well as walking drills  Co-intervention: Home- based: Psychological support; and dietary and self- management advice through telephone or a video conference. Hospital- based: participate in a multidisciplinary maintenance program  including physiotherapy, | Active control:  CG1: hospital- based PR program  CG2: Usual care | Home-based: sessions with remote monitoring  Hospital-based: in- person, twice weekly. | 12  months | 6MWT,  Physical activity, HRQoL: SGRQ  and CAT, exacerbation and hospitalizatio n risk | The home-based maintenance tele- rehabilitation was as effective as hospital-based, outpatient, and superior to usual care in terms of reducing the risk for acute exacerbation of COPD and hospitalizations, while preserving the functional and HRQoL benefits of a primary PR program over a period of 12 months | Not reported |

|  |  |  |  |  | dietary and psychological advice |  |  |  |  |  |  |
| --- | --- | --- | --- | --- | --- | --- | --- | --- | --- | --- | --- |
| Wang 2014  (49) | RCT | HBG: 71.4 ± 1.9  CG: 71.9 ± 2.7 | HBG: 63.5 ±  7.0  CG: 54.2 ± 6.7 | 30 | Endurance training: walk at a speed following the tempo of music on the mobile phone. Exercise program was installed into phone  Co-intervention: none | Usual care | Remotely, phone calls and in-person every 4 weeks | 6 months | Endurance walking exercise with constant intensity from ISWT,  measurement s of muscle strength, IL-6, IL-8 and TNF-  α | Improve at exercise capacity, strength of limb muscles and reducing inflammatory biomarkers | Not reported |
| Wewel 2007 (19) | One group pre- and post-test | 65 ± 9 | 34.8 ± 5.0 | 21 | Raise home-based activity (target: 3 times of 15 min daily at 75% of maximum dyspnea). 3 times daily 15 min of raised walking activity producing the dyspnea which had been  elicited at 75% of the individual 6MWT upon visit  Co-intervention: none | None | Regular phone calls | 2 weeks | Exercise capacity: 6MWT, HRQoL: SF-36  and SGRQ | There was increase exercise capacity and quality of life | Not reported |
| Wijkstra 1996a;  1994 (44,  45)  (Two reports from the same study) | RCT | HBG: 64 ± 5  CG: 62 ± 5 | HBG: 44 ± 11  CG: 45 ± 9 | 43 | General program: relaxation exercises, breathing retraining, upper limb training, target flow inspiratory muscle training, and exercise training Endurance training: started at 60% of their maximum workload with progressive increase in the time-duration in the cycle ergometer test | Usual care | In-person, weekly | 12 weeks | Wijkstra 1996a:  Exercise capacity: 6MWT and incremental symptom- limited cycle ergometer test. Lactate production, VO2max and dyspnoea | Wijkstra 1996a: The experimental group showed a significant increase in 6MWT, maximum workload, in the oxygen consumption (VO2), lactate level and dyspnea compared with the control group  Wijkstra 1994: highly significant improvement in the rehabilitation group compared to the control group for the dimensions dyspnoea, emotion, and mastery (CRQ).. The exercise tolerance improved significantly in the rehabilitation  group compared to the control group. | Not reported |

|  |  |  |  |  | Upper limb was according to the principles of proprioceptive neuromuscular facilitation  Co-intervention: education sessions |  |  |  | Wijkstra 1994: HRQoL (CRQ);  Exercise tolerance (cycle ergometer test) |  |  |
| --- | --- | --- | --- | --- | --- | --- | --- | --- | --- | --- | --- |
| Wijkstra 1996b;1995 (20, 21)  (Two report from the same study) | RCT | HBG-A: 62.3 ±  5.1  HBG-B: 64.0 ±  6.2  CG-C: 61.9 ± 3.6 | HBG-A: 43.2 ±  11.3  HBG-B: 45.3 ±  12.3  GC-C: 42.9 ±  8.7 | 36 | Endurance training: upper limb training, exercise training at 60% of Wmax in the bicycle ergometer test. The time span was gradually extended to 12 min and the work load to a maximum of 75% of the Wmax. Twice a day for 30 min during the first 3 months; thereafter, once a day for 30 min  Co-intervention: relaxation exercises; breathing training | No rehabilitation | Supervised by a multidisciplinary team during the rehabilitation program at home.  HBG- A once week supervision at follow up.  HBG-B monthly supervision at follow up  CG-C no rehabilitation | 3 months of PR and 18  months of maintena nce | Wijkstra 1996b: Lung function; Bicycle ergometer test; 6MWD; Strength and endurance capacity of the inspiratory muscles.  Wijkstra 1995: HRQoL (CRQ);  6MWD; Lung function | Wijkstra 1996b: Study showed that home rehabilitation neither improves exercise tolerance nor cardiocirculatory parameters. FEV 1, dyspnea score, 6MWD, and other variables were not significantly different between the three groups. Wijkstra 1995: Improvements in quality of life were found in patients in groups A and B, but not in those in group C compared with baseline, but these only reached significance in group B at all time points. Patients in group B had a higher quality of life than those in group C only at three and 12 months. There was a decrease in both 6MWD (at 12 and 18 months) and inspiratory vital capacity (at three 12, and 18 months) in patients in group C compared with the baseline measurement. Between groups analysis showed no differences for 6MWD distance, FEV1, and inspiratory vital capacity at follow up. | Not reported |
| Zanaboni 2013 (43) | One group Pilot study | 54.0 [51.0–  56.8] | 38.5 [34.0–  61.3] | 10 | Endurance training: at treadmill with intensity determined by Borg scale, oxygen saturation and heart rate  Patients received an exercise station including a treadmill, a | None | Remotely by videoconference, weekly | 24  months | Hospital admissions. long-term exercise maintenance; adherence to the exercise program;  hospital | There was a 27% difference in the COPD-related hospital costs as consequences of fewer accesses and shorter length-of-stay, despite this difference was not significant. After more than one year since enrolment, all participants continue to participate actively, and no drop-outs  have occurred | Not reported |

|  |  |  |  |  | pulse oximeter and a tablet computer  Co-intervention: web- based education, self- management platform |  |  |  | length-of- stay, healthcare costs; quality of life |  |  |
| --- | --- | --- | --- | --- | --- | --- | --- | --- | --- | --- | --- |

Abbreviations: FEV1, forced expiratory volume; RCT, randomized controlled trial; HBG, home-based group; CG, control group; Telerehab, telerehabilitation;; 6MWT, six-minute walk test; 6MWD, six-minute walk distance; 6MTD, six-minute treadmill distance; HRQoL, health-related quality of life; CRQ, chronic respiratory questionnaire; PR, pulmonary rehabilitation; PA, physical activity; COPD, chronic obstructive pulmonary disease; CAT, COPD assessment test; ISWT, incremental shuttle walk test; ESWT, endurance shuttle walk test; MRC, medical research council; SGRQ, St. George’s respiratory questionnaire, LCADL, London chest activity of daily living; AQ-20, airways questionnaire 20; ADL, activity daily living; SF-36, short-form health survey; VO2, oxygen consumption; IL, interleukin; Wmáx, maximal workload.
