# Supplementary material for: Embedding Pulmonary Rehabilitation for Chronic Obstructive Pulmonary Disease in the Home and Community Setting: A Rapid Review
Source: Front Rehabil Sci. 2022 Mar 30;3:780736. doi: 10.3389/fresc.2022.780736 (PMC9397727; doi:10.3389/fresc.2022.780736)
Supplement: Supplementary file 3 [file Table_3.docx]

**Supplemental Table 3.** Characteristics of included studies on community-based pulmonary rehabilitation

| **Study** | **Design** | **Age (Years)** | **FEV1 (% predicted)** | **Sample**  **size** | **Community-based**  **exercise program** | **Comparison**  **intervention** | **Program**  **supervision** | **Local** | **Duration** | **Outcome** | **Effects** | **Barriers and**  **facilitators** |
| --- | --- | --- | --- | --- | --- | --- | --- | --- | --- | --- | --- | --- |
| Amin 2014 (93) | RCT | CBG: 66.8 ± 8.1  CG: 72 ± 10.1 | CBG: 63.6 ± 7.6  CG: 60.8 ± 10.9 | 19 | Endurance training: continuous aerobic exercise during 30 min in the target heart rate range based on maximal exercise incremental test on a treadmill ergometer Resistance training  Co-intervention: nutritional counseling | Usual care | In-person, weekly | Health clubs | 12 weeks | Session compliance; adherence; Exercise capacity: treadmill endurance time; muscle strength; dyspnea; HRQoL: SGRQ | Compliance was 94% and adherence was 83%. CBG group increased muscle strength and endurance time and reduced dyspnea compared with CG. HRQoL was not significantly changed | Not reported |
| Arbillaga-Etxarri 2018 (91) | RCT | CBG: 69 ± 9  CG: 69 ± 8 | CBG: 56 ± 17  CG: 57 ± 18 | 407 | Endurance training: walk in an urban circuit at a pace reaching a dyspnea Borg scale between 4 and 6  Phone text messages every 2 weeks with educational or motivational messages  A pedometer and a personalized calendar to monitor their physical activity  Co-intervention:  behavioural | Active control: counseling to perform physical activity of moderate intensity 30  min 5 days/ week | Remotely, phone calls every 3 months | Street, square, gardens | 12  months | Physical activity (step/day); 6MWT; Anxiety and depression; HRQoL: CAT;  Health status: CCQ; Body composition: BMI, FFMI;  Exacerbation | There was an increase in physical activity in the experimental group.  However, it was ineffective in the full population including unwilling and self-reportd non adherent patients. The intervention had no effect on severe COPD exacerbations, 6MWT, body composition, HRQoL, anxiety, or depression Adverse events: lower extremity muscle pain during walks in patients of usual care group | Not reported |

|  |  |  |  |  | motivational strategies by text messages and pedometer |  |  |  |  |  |  |  |
| --- | --- | --- | --- | --- | --- | --- | --- | --- | --- | --- | --- | --- |
| Beauchamp 2013 (92) | One-group longitudinal pre- and post- test, maintenance program | 66.8 ± 7.8 | 45.1 ± 18.8 | 29 | Endurance training: walking along a designated track with rests as needed, cycling or treadmill up to 1 hour  Resistance training: free weights and ‘wall climbing’ for upper extremity; mini-squats, step- ups, and resistance bands for lower extremity; optional exercises for upper and lower extremity using gym equipment (e.g., chest press, leg extension/curls, leg press)  Co-intervention: none | None | In-person, all sessions | Community recreation center | 12  months | Exercise capacity: 6MWT and sit to stand test; Self- efficacy; Physical Activity Scale for the Elderly; HRQoL: CRQ;  Adherence | Mean compliance to the program was 70%.  Significant difference in the exercise capacity, self-efficacy and HRQoL total were observed at 6- months and 1-year after PR. Community maintenance program preserved exercise capacity and HRQoL. Adverse events: no adverse events were related to exercise program | Main barriers: medical problems, travel difficulties and social problems |

| Cambach 1997  (89) | RCT and cross-over design | 62 ± 7 | 59.3 ± 19 | 26 | 3 sessions/week Endurance training: performed on a cycle ergometer (60-75% Wmáx;  duration increased from 3 to 12 min), on a rowing machine (60% or more of HRmáx) and by stair-walking (60% or more of HRmáx).  Additionally, recreational activities to maintain benefits after rehabilitation (swimming, cycling and hockey at 60% or more of HRmáx)  Co-intervention: Education sessions and relaxation techniques | Drug treatment | In-pearson, all sessions | Community | 3 months | HRQoL (CRDQ);  6MWT; Exercise tolerance (incremental, endurance and submaximal cycle ergometer test) | Within-group analysis for the patients first receiving the rehabilitation program and then the control condition showed that the cycling endurance time, HR during cycling, and the walking distance improved significantly during the rehabilitation period compared to the control period. | Not reported |
| --- | --- | --- | --- | --- | --- | --- | --- | --- | --- | --- | --- | --- |

| Cecins 2017  (96) | Prospective observational study | 72 ± 9 | 54 ± 24 | 251 | 2 days/ week Endurance training: 20–30 min of ground-based walking prescribed at 80% of the average speed achieved on the 6MWT  Where necessary, interval training was used to maintain oxygen saturation (SpO2) ≥ 85%  Co-intervention: education sessions and self- management | None | In-pearson all sessions | Community | 8 weeks  and 12 months follow-up | Exercise capacity (6MWT); HRQoL  (CRQ); Hospital admissions | Improvements were demonstrated in 6MWD and total CRQ score Fewer participants had a respiratory-related hospital admission following the program (12 months) | Not reported |
| --- | --- | --- | --- | --- | --- | --- | --- | --- | --- | --- | --- | --- |
| Desveaux 2014  (78) | Qualitative study | 68 | 44 | 12 | Endurance training: not specified  Co-intervention: none | None | In-person, all sessions | Community recreation center | 12  months | Participant experiences (barriers and facilitators) | Participants expressed more benefits (improved function and HRQoL) than barriers. Barriers included exacerbations, fatigue, access to transportation, and weather | Not reported |
| Doyle 2017 (79) | One-group longitudinal pre- and post- test | Not provided | Not provided | 195 | Followed the AACVPR guidelines for pulmonary rehabilitation  Co-intervention: education sessions | None | In-person, all sessions | Community health center and critical assess rural hospital | 12 weeks | Dyspnea: mMRC; Exercise capacity: 6MWT; HRQoL:SGRQ;  Inspiratory muscle strength; Adherence | Mean improvements for all outcomes were highly significant.  57% completed the program | Not reported |

| Elliot 2004 (80) | RCT  Maintenance program | CBG: 62.5 ± 2.1  HBG: 66.4 ± 2.1 | CBG: 42.7 ± 5.9  HBG: 46 ± 3.9 | 31 | Walking, general exercises or low intensity circuit with weights; stretch  Co-intervention: education sessions | Active control: Circuit strength training for upper, lower and abdominal; aerobic training: treadmill and corridor walking, cycling; stretch | In-person, all sessions | Community Physiotherapy Services of the Health Department of Western Australia | 12  months | Exercise capacity: 6MWT; HRQoL: CRQ | At 3 months, there was a significant improvement in HRQoL in the Hospital group and in the Community group, but the difference between the groups was not significant. A 3-month community-based exercise program did not improve 6MWT | Not reported |
| --- | --- | --- | --- | --- | --- | --- | --- | --- | --- | --- | --- | --- |
| Effing 2011 (81) | RCT | CBG: 62.9 ± 8.1  CG: 63.9 ± 7.8 | CBG: 49.6 ± 14.2  CG: 50.5 ± 17 | 153 | Endurance training: bicycling, walking and climbing stairs Resistance training for upper and lower limbs  Co-intervention: pharmacological support, smoking cessation program and self- management sessions | Self- management sessions without exercise | In-person all session at the community. The sessions at home were unsupervised | Community health center | 11  months | Exercise capacity: ISWT and ESWT; HRQoL:CRQ-SAS;  Health status: CCQ; Anxiety and depression; Fat free mass; Physical activity | Significant between- group differences in ISWT and daily activity were found in favour of the experimental group. Over the 12-month period a significant difference of the CRQ – dyspnea score and a non-significant difference of the ESWT was found. No differences were found in the other CRQ-  components, anxiety and depression scores and percentage of fat free mass  Adverse events: no adverse events were related to exercise training | Not reported |

| Fastenau 2020  (82) | RCT | CBG: 62.4 ± 9.1  CG: 62.6 ± 10.8 | CBG: 74.4 ± 14.8  CG: 74 ± 12.2 | 90 | Endurance training and/or interval training: walking speed on the treadmill at 75% or higher of the results of the 6MWT, ratings of perceived exertion and dyspnoea of five and higher on the modified Borg- scale  Resistance training: upper and lower limbs at least 60- 80% of 1 RM  Breathing exercise: active expiration, slow and deep breathing, pursed lips breathing, relaxation therapy and diaphragmatic breathing  Co-intervention: respiratory exercises and additional training session at home (i.e. walking or  cycling) | Active control: perform low- intensity exercise training on a treadmill or stationary cycling (Dyspnea Borg modified scale  < 2). There were no breathing exercises or resistance training. | In-person, all sessions | Physiotherapy setting in primary care | 6 months | Exercise capacity: 6MWT; Dyspnea: MRC; HRQoL: CCQ, and CRQ;  muscle strength; Daily physical activity | The results indicate that the intervention is particularly effective in improving exercise capacity and strength, but not in dyspnea, HRQoL and daily physical activity | Not reported |
| --- | --- | --- | --- | --- | --- | --- | --- | --- | --- | --- | --- | --- |
| Godtfredsen 2018 (83) | One-group longitudinal pre- and post- test | 68.1 ± 11.1 | 53.5 ± 25.2 | 581 | 6-12 weeks, 2 days/week Endurance training: aerobic exercise  Resistance training Breathing exercises  Co-intervention: education,  nutritional and | None | In-person, all sessions | Primary care setting | 6-12  weeks | Exercise capacity: 6MWT; HRQoL:  Generic 15-item questionnaire 15D | Effects on exercise capacity and HRQoL that were clinically meaningful | Not reported |

|  |  |  |  |  | psychosocial support |  |  |  |  |  |  |  |
| --- | --- | --- | --- | --- | --- | --- | --- | --- | --- | --- | --- | --- |
| Golmohammadi 2004 (84) | Cost- effectiveness study | 68.5 ± 8.5 | 55.1 ± 21.9 | 210 | Endurance: aerobic and interval training upper extremity conditioning and inspiratory muscle conditioning  Co-intervention: education, psychosocial support and nutritional counseling | None | In-person, all sessions | Community health center | 6 weeks  or 8 weeks, and follow-up 12  months | Direct costs; HRQoL: SGRQ | Over one-year, CBPR was associated with decreased health service utilization, reduced direct costs and improved health status | Facilitators: less distance to travel; easier  parking; a sense of community among the patients |
| Jones 2002 (85) | One-group longitudinal pre- and post- test  Pilot study | 50 - 81 | 43.4 (24 – 63) | 14 | It doesn't specify which modalities, it just mentions 'exercises'  Co-intervention: education sessions | None | In-person, all sessions | Community health clinic | 12  months | Exercise capacity: ISWT; HRQoL: CRDQ; | There was a mean 50- metre improvement in ISWT after the program, an increase of 37%, but this returned to baseline over the next 12 months. Sustained improvements were found in health status measures. The CRDQ showed marked improvements: the mean total score rose by 20 points | Facilitators: less distance to travel; easier  parking; a sense of community among the patients |

| Machado 2019  (77) | Quasi- experimental Pilot study | CBG: 68.6 ± 7.4  CG: 65.8 ± 8.8 | CBG: 51.5 ± 21.6  CG: 55 ± 21.5 | 23 | Endurance training: walk, stepping or cycling at 60-80% of maximum estimated heart rate and symptoms of dyspnea and perceived exertion between 4 and 6 at the modified Borg scale  Breath retraining, airway clearance techniques, thoracic mobility, flexibility and stretch exercises  Co-intervention: psychoeducational support | Usual care | All sessions were supervised | School of Health Sciences (University) | 3 weeks | Dyspnea: mMRC; quadriceps muscle strength; functionality: 5- STS; HRQoL: CAT;  Hospitalization | The CBG presented improvements on quadriceps muscle strength, HRQoL and dyspnoea at rest. No improvements for functionality. None of the patients, either in the CBG or in the CG, needed to be hospitalized or use of the healthcare services during the 3 weeks of intervention  Adverse events: no adverse events related to exercise training | Not reported |
| --- | --- | --- | --- | --- | --- | --- | --- | --- | --- | --- | --- | --- |
| Meshe 2020  (95) | Qualitative study | 74.3 | 66 | 12 | 2 days p/week Exercise training includes breathing exercises, 24- seated warm-up and cool-down exercises, 5 cardiovascular and 12 resistance- training exercises  Co-intervention: none | None | All sessions were supervised | Community recreation centre | 3 months | Benefits, barriers and facilitators of adherence to a community- based pulmonary rehabilitation program | Facilitators: ease of access, perceived benefits and convenient program components, being a retiree, social support and seasons. Barriers: poor physical health, family commitments and transport difficulties | Facilitators: ease of access, perceived benefits and convenient program components, being a retiree, social support and seasons. Barriers: poor physical health, family commitments and transport  difficulties |

| Varas 2018 (90) | RCT | CBG: 69.5 ± 7.4  CG: 64.8 ± 9.1 | CBG: 45.8 ± 16.5  CG: 52.3 ± 15.7 | 40 | Endurance: walk for 30–60 min (in  cycles of 15–20 min) at speeds based on the last level completed of ISWT. Five initial sessions of respiratory physiotherapy (e.g.ventilation techniques, bronchial clearance)  Co-intervention: none | Usual care | Weekly through a telephone call | Primary health-care centre | 8 weeks and follow-up 12  months | Exercise capacity: ESWT; self- reported physical activity; pedometer: number of steps; HRQoL: SGRQ;  exacerbation | The CBG showed improvements in ESWT times and distance, number of steps, PA scores and SGRQ scores. These results remained evident after 3 and 12 months. There were no differences between the groups in the exacerbation | Not reported |
| --- | --- | --- | --- | --- | --- | --- | --- | --- | --- | --- | --- | --- |
| Vest 2011 (86) | RCT | CBG: 73.5 (49.3  – 87.4)  CG: 71.6 (50.4 –  89.8) | CBG: 44 (19.3 –  68.3)  CG: 41.2 (21.3 –  102.5) | 123 | Endurance: walking and cycle training with a velocity at 85% of predicted maximal oxygen uptake in ESWT  Co-intervention: education sessions | Conventional outpatient hospital-based exercise program | In-person all session | Primary health-care centre | 7 weeks and follow-up 3 months | Exercise capacity: ESWT; HRQoL:  SGRQ; adherence | Improvements in ESWT and HRQoL were found for both groups, however, the improvement of ESWT was greater in the secondary care setting. Adherence was high for CBG  Adverse events: no adverse events were related to exercise training | Not reported |

| Zakrisson 2013  (87) | Qualitative study | 66 | None | 20 | Endurance training: aerobic exercises; Resistance training; Breathing, coughing, relaxation techniques  Co-intervention: education sessions | None | Not described | Primary health-care centre | 6 weeks | Experience of a program of pulmonary rehabilitation from the perspective of the next of kin | Despite the program, the next of kin felt that their lives were overshadowed by the illness. Although somewhat insufficient in relation to their needs and expectations, the program had positive outcomes for next of kin at the two-year follow- up. It enabled the couples to talk about the difficulties, and the next of kin sensed greater togetherness in the relationship as well as a relief in their personal burden. | Not reported |
| --- | --- | --- | --- | --- | --- | --- | --- | --- | --- | --- | --- | --- |

| Zwerink 2014  and 2016 (88,  94) | RCT | CBG: 63.1 ± 8.1  CG: 64.1 ± 7.7 | CBG: 49.6 ± 14.2  CG: 50.5 ± 17 | 153 | Endurance training: bicycling, walking and climbing stairs Resistance training: lifting weights  Co-intervention: pharmacological support, smoking cessation program and self- management sessions | Active control. Participants received self- management program | In-person, all sessions at community health center. The sessions at home were unsupervised | Community health center | 12  months and 24 months of follow-up | Exercise capacity: ISWT and ESWT;  Daily physical activity; HRQoL: CRQ; Health status: CCQ; Anxiety and depression (HADS);  Adherence; Costs per patient | There was a between- group difference in maximal exercise capacity at 12 months but not after 24 months. The between-group difference in daily physical activity was maintained after 24 months. Improvement was also found on CRQ dyspnea score but not on other CRQ domains, CCQ and HADS.  Adherence was high for both groups at 24 months follow-up  The ICER for an additional patient prevented from deteriorating at least  47.5 meters on the ISWT was €6257. The ICER for an additional patient with a clinically relevant improvement (≥ 500 steps/day) in physical activity was €1564, and the ICER for an additional quality- adjusted life year was  €10950 | Not reported |
| --- | --- | --- | --- | --- | --- | --- | --- | --- | --- | --- | --- | --- |

Abbreviations: FEV1, forced expiratory volume; RCT, randomized controlled trial; CBG, community-based group; CG, control group; 6MWT, six-minute walk test; HRQoL, health-related quality of life; SGRQ, St. George’s respiratory questionnaire; CRQ, chronic respiratory questionnaire; CCQ, clinical COPD questionnaire; MRC, medical research council; ESWT, endurance shuttle walk test; ISWT, incremental shuttle walk test; HADS, hospital anxiety and depression scale; CRQ-SAS, chronic respiratory questionnaire self-administered standardized; CAT, COPD assessment test; mMRC, modified medical research council; PA, physical activity; PR, pulmonary rehabilitation; 5-STS, 5 repetition sit-to-stand test; BMI, body mass index; FFMI, fat free mass index; ICER, incremental cost-effectiveness ratio; HR, heart rate.
